# Robust and prototypical immune responses toward influenza vaccines in the high-risk group of Indigenous Australians

**DOI:** 10.1073/pnas.2109388118

**Published:** 2021-10-04

**Authors:** Luca Hensen, Thi H. O. Nguyen, Louise C. Rowntree, Timon Damelang, Marios Koutsakos, Malet Aban, Aeron Hurt, Kim L. Harland, Maria Auladell, Carolien E. van de Sandt, Anngie Everitt, Cath Blacker, Damian A. Oyong, Jessica R. Loughland, Jessica R. Webb, Bruce D. Wines, P. Mark Hogarth, Katie L. Flanagan, Magdalena Plebanski, Adam Wheatley, Amy W. Chung, Stephen J. Kent, Adrian Miller, E. Bridie Clemens, Peter C. Doherty, Jane Nelson, Jane Davies, Steven Y. C. Tong, Katherine Kedzierska

**Affiliations:** ^a^Department of Microbiology and Immunology, University of Melbourne, The Peter Doherty Institute for Infection and Immunity, Parkville, VIC 3000, Australia;; ^b^World Health Organization Collaborating Centre for Reference and Research on Influenza, The Peter Doherty Institute for Infection and Immunity, Melbourne, VIC 3000, Australia;; ^c^Department of Hematopoiesis, Sanquin Research and Landsteiner Laboratory, Amsterdam UMC, University of Amsterdam, 1066 CX Amsterdam, The Netherlands;; ^d^Menzies School of Health Research, Charles Darwin University, Darwin, NT 0810, Australia;; ^e^Center for Global Infectious Disease Research (CGIDR), Seattle Children's Research Institute, Seattle, WA 98109;; ^f^Immunology Group, QIMR Berghofer Medical Research Institute, Brisbane, QLD 4029, Australia;; ^g^Immune Therapies Laboratory, Burnet Institute, Melbourne, VIC 3084, Australia,; ^h^Department of Immunology and Pathology, Central Clinical School, Monash University, Melbourne, VIC 3010, Australia;; ^i^Department of Clinical Pathology, The University of Melbourne, Parkville, VIC 3010, Australia;; ^j^Tasmanian Vaccine Trial Centre, Clifford Craig Foundation, Launceston General Hospital, Launceston, TAS 7250, Australia;; ^k^School of Health Sciences and School of Medicine, University of Tasmania, Launceston, TAS 7248, Australia;; ^l^Department of Immunology and Pathology, Monash University, Melbourne, VIC 3800, Australia;; ^m^School of Health and Biomedical Science, Royal Melbourne Institute of Technology, Melbourne, VIC 3000, Australia;; ^n^Australian Research Council Centre of Excellence in Convergent Bio-Nano Science and Technology, University of Melbourne, Melbourne, VIC 3010, Australia;; ^o^Infectious Diseases Department, Melbourne Sexual Health Centre, Alfred Health, Central Clinical School, Monash University, Melbourne, VIC 3053, Australia;; ^p^Indigenous Engagement, Central Queensland University, Townsville, QLD 4810, Australia;; ^q^Department of Immunology, St. Jude Children’s Research Hospital, Memphis, TN 38105;; ^r^Department of Infectious Diseases, Royal Darwin Hospital, Darwin, NT 0810, Australia;; ^s^Victorian Infectious Diseases Service, The Royal Melbourne Hospital and The University of Melbourne, The Peter Doherty Institute for Infection and Immunity, Parkville, VIC 3000, Australia

**Keywords:** influenza, Indigenous people, antibodies, B cells, follicular T helper cells

## Abstract

Morbidity and mortality rates from seasonal and pandemic influenza occur disproportionately in high-risk groups, including Indigenous people globally. Although vaccination against influenza is recommended for those most at risk, studies on immune responses elicited by seasonal vaccines in Indigenous populations are largely missing, with no data available for Indigenous Australians and only one report published on antibody responses in Indigenous Canadians. We recruited 78 Indigenous and 84 non-Indigenous Australians vaccinated with the quadrivalent influenza vaccine into the Looking into InFluenza T cell immunity - Vaccination cohort study and collected blood to define baseline, early (day 7), and memory (day 28) immune responses. We performed in-depth analyses of T and B cell activation, formation of memory B cells, and antibody profiles and investigated host factors that could contribute to vaccine responses. We found activation profiles of circulating T follicular helper type-1 cells at the early stage correlated strongly with the total change in antibody titers induced by vaccination. Formation of influenza-specific hemagglutinin-binding memory B cells was significantly higher in seroconverters compared with nonseroconverters. In-depth antibody characterization revealed a reduction in immunoglobulin G3 before and after vaccination in the Indigenous Australian population, potentially linked to the increased frequency of the G3m21* allotype. Overall, our data provide evidence that Indigenous populations elicit robust, broad, and prototypical immune responses following immunization with seasonal inactivated influenza vaccines. Our work strongly supports the recommendation of influenza vaccination to protect Indigenous populations from severe seasonal influenza virus infections and their subsequent complications.

## Significance

Indigenous populations worldwide are highly susceptible to influenza virus infections. Vaccination with inactivated virus is highly recommended to protect Indigenous populations, including Indigenous Australians. There is no study to date that assessed immune responses induced by the inactivated seasonal influenza vaccine in the Indigenous population. Vaccine recommendations are thus based on data generated for non-Indigenous populations and might not be representative for Indigenous people. We found robust antibody responses to influenza vaccination induced in Indigenous Australians, with activation profiles of cT_FH_1 cells at the acute response strongly correlating with total change of antibody vaccine titers induced by vaccination. Our work strongly supports the recommendation of influenza vaccination to protect Indigenous populations from severe seasonal influenza virus infections and subsequent complications.

Influenza is a significant respiratory viral infection that causes a serious burden of disease. High morbidity and mortality rates from seasonal and pandemic influenza occur disproportionately in specific high-risk population groups, including children, the elderly, pregnant women, Indigenous people globally, and individuals with underlying comorbidities such as diabetes, immunosuppression, and lung and heart disease (1–3). Currently, antibody-based influenza vaccines targeting highly variable hemagglutinin (HA) and neuraminidase (NA) surface glycoproteins are the most effective way to combat seasonal infections. The inactivated influenza vaccine contains glycoproteins corresponding to the circulating A/H3N2 and A/H1N1 strains and one B strain from either the Victoria or Yamagata lineage (in trivalent/TIV vaccines) or both B lineage strains (in quadrivalent/QIV vaccines). Antigenic drift necessitates annual updates of the vaccine components to warrant protection, and despite this the overall vaccine effectiveness can vary vastly from −7 to 75% (4). Vaccine effectiveness not only differs between seasons but also between vaccine components, with H3N2 showing the lowest overall vaccine effectiveness and H1N1pdm09 (pH1N1) the highest (5). Several factors such as preexisting immunity, immunosenescence, and vaccine strain mismatch can influence vaccine effectiveness (6). Genetic factors such as HLA polymorphisms contributing to differences in HLA-II expression are associated with stronger or weaker vaccine responses (7). For example, individuals expressing HLA-DRB1*11:04 showed high titers postvaccination whereas HLA-DRB1*13:01 showed reduced antibody titers postvaccination (7).

Our understanding of why some individuals fail to establish a protective immune response after influenza vaccination is still very limited. To determine which factors shape the immune response postvaccination, we and others have identified cellular and humoral responses that correlate with robust immune responses to influenza vaccination (8, 9). Importantly, 7 d postvaccination an increase in ICOS^+^CXCR3^+^CXCR5^+^CD4^+^ circulating T follicular helper 1 (cT_FH_1) cells was observed that correlated with antibody-secreting cells (ASCs) and rises in antibody titers (9, 10).

Indigenous populations experience higher rates of infections with a range of pathogens including tuberculosis (11) and influenza (12). Notification and hospitalization rates of seasonal influenza virus infections are 1.5 to 8.6 times and 1.2 to 4.3 times, respectively, higher in Indigenous compared with non-Indigenous Australians (12). With social determinants of health and comorbidities contributing to a higher disease burden (13), one key strategy proposed to improve health outcomes for Indigenous populations is immunization (14). However, only a few studies to date have examined viral immunity in Indigenous populations and most of our knowledge is based on studies in non-Indigenous populations. We have revealed host variations in HLA profiles in Indigenous populations (15, 16), suggesting that differences in HLA or other genetic factors might impact influenza vaccine responses in Indigenous Australians. Despite national funding, vaccination rates still remain low in Indigenous communities (17). A recent study from Menzies et al. revealed that more than 50% of unvaccinated Indigenous Australians stated that the “flu” vaccine would not be effective (18). To date, there are no published data to define immune responses to influenza vaccines in Indigenous Australians, while globally only one study assessed antibody responses following adjuvanted pH1N1 influenza immunization in Indigenous Canadians and showed comparable antibody levels pre- and postvaccination (19). Determining the immunological response to influenza vaccination in high-risk Indigenous populations can therefore provide a stronger scientific basis for influenza recommendations, which if appropriately communicated may increase vaccine uptake.

In this study, we recruited Indigenous and non-Indigenous Australians vaccinated with the QIV between 2016 and 2018 and assessed their immunity pre- and postvaccination. We performed in-depth analyses of T and B cell activation, memory B cell formation, and antibody profiles as well as investigating host factors that could contribute to vaccine responses. Our study clearly demonstrates that Indigenous Australians mount effective and prototypical immune responses to the inactivated influenza vaccine and thus provides an immunological basis to support current vaccine recommendations in Indigenous populations.

## Results

### Recruitment of LIFT-V Cohort.

**Fig. 1.**
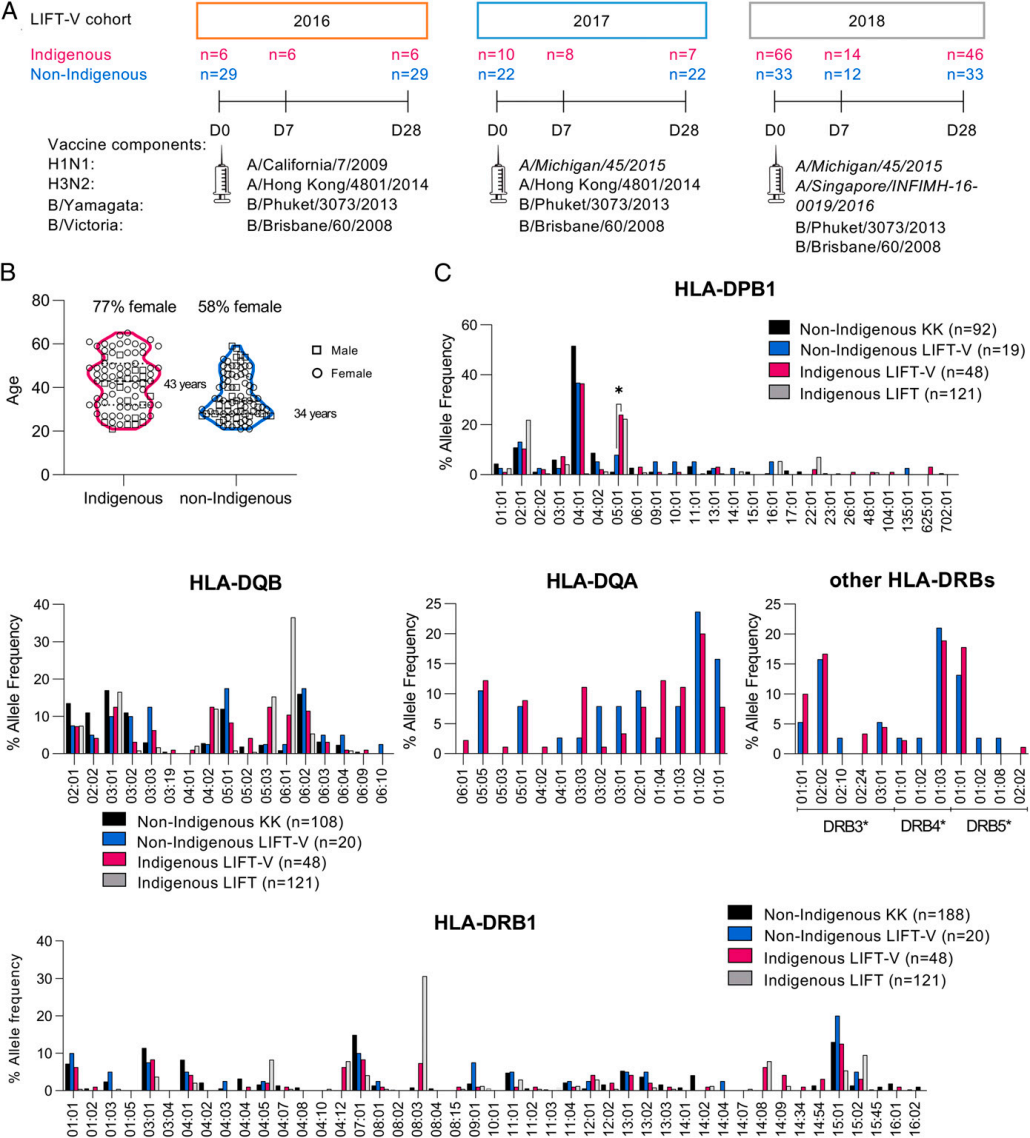
Indigenous Australians display broad and distinct HLA-II profiles. (*A*) Study design, vaccine composition, and participants of the LIFT-V cohort between 2016 and 2018. (*B*) Age distribution of Indigenous and non-Indigenous participants of the LIFT-V cohort. (*C*) Allele frequencies of HLA-II alleles in non-Indigenous and Indigenous participants of the LIFT-V cohort. Allele frequencies from additional cohorts (non-Indigenous individuals and Indigenous LIFT cohort) were included from previous HLA datasets (15). Differential distribution of HLA-allele-expressing donors was analyzed between Indigenous and non-Indigenous LIFT-V donors using χ^2^ test (*P < 0.05).

To assess immunological responses toward the QIV, Indigenous (*n* = 78) and non-Indigenous (*n* = 84) participants were recruited between 2016 and 2018 into the Looking into InFluenza T cell immunity - Vaccination (LIFT-V) cohort (Fig. 1*A*). The vaccine H1N1 strain changed from 2016 (A/California/7/2009) to 2017 (A/Michigan/45/2015) and the H3N2 strain from 2017 (A/Hong Kong/4801/2014) to 2018 (A/Singapore/INFIMH-16-0019/2016). Blood and serum samples were collected prior to vaccination (day 0, d0) and postvaccination (days 7 and 28, d7 and d28). While only a limited number of donors was sampled at the early bleed on d7 (34% of Indigenous and 14% of non-Indigenous participants), a high frequency of sampling was achieved on d28 (72% and 100%, respectively). Of the Indigenous cohort, 76% of participants were female, whereas 58% of non-Indigenous participants were female. The median age was 43 y (range 21 to 65 y) in the Indigenous cohort and 34 y (range 21 to 59 y) in the non-Indigenous cohort (Fig. 1*B*).

### Diverse HLA Class II Expression in Indigenous and Non-Indigenous Australians.

Previous studies have shown different patterns of HLA profiles between Indigenous and non-Indigenous Australians (15). Given that HLA class II (HLA-II) expression is known to impact influenza vaccine responses (7), we assessed whether the HLA-II profiles differed between our Indigenous and non-Indigenous cohorts and whether the previously published HLA-II alleles associated with altered influenza antibody responses (7) were prominent in our cohorts. In contrast to the previously reported differences for HLA-I profiles (15), the HLA-II profiles (DPB1, DQA, DQB, and DRB1/3/4/5) were similar between Indigenous and non-Indigenous LIFT-V cohorts and also comparable to our larger dataset of Indigenous (LIFT) and non-Indigenous (KK) cohorts (Fig. 1*C*). Both of our Indigenous cohorts (LIFT and LIFT-V) showed similar HLA-II profiles, with high allelic frequencies of HLA-DQB*04:02 (12% in LIFT and 12.5% in LIFT-V), 05:03 (15.3% in LIFT and 12.5% in LIFT-V), 06:01 (36.5% in LIFT and 10.4% in LIFT-V), DRB1*04:12 (7.9% in LIFT and 6.3% in LIFT-V), 08:03 (30.6% in LIFT and 7.3% in LIFT-V), and DPB1*05:01 (22.3% in LIFT and 24% in LIFT-V) (Fig. 1*C*). Of note, previously published alleles associated with a lower (DRB1*07, DQB1*03:03) (7, 20) or stronger (DRB1*13, DRB3*0X, DQB1*06:03-9/14) (7, 20) antibody response toward the influenza vaccine were not significantly different in the Indigenous cohort compared with the non-Indigenous group. However, we detected a significantly increased frequency of HLA-DPB1*05:01-expressing individuals in Indigenous Australians compared with non-Indigenous Australians in the LIFT-V cohort. Analyzing all the genotyped donors available in our laboratory (Non-Indigenous KK & LIFT-V vs. Indigenous LIFT & LIFT-V), totaling 280 individuals, we observed the same results as we found in the analysis of the vaccine cohorts alone.

### Robust Antibody Responses to Influenza Vaccination Induced in Indigenous Australians.

**Fig. 2.**
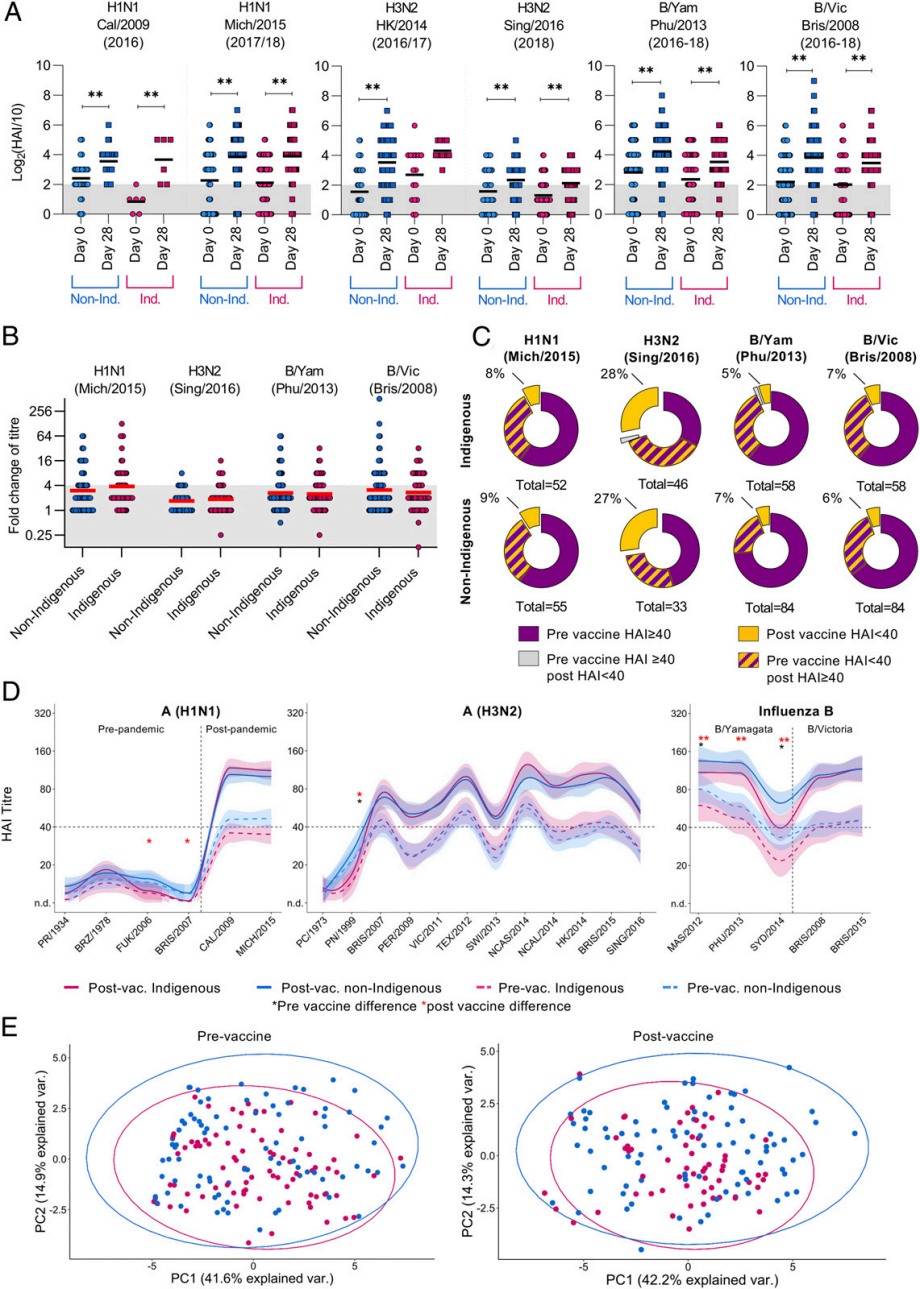
Comparable HAI responses between Indigenous and non-Indigenous donors against vaccine strains and previously circulating viruses. (*A*) Serum antibodies were determined by HAI assay prior to vaccination (day 0) and postvaccination (22 to 85 d post vaccination) against all four vaccine components. Bold line indicates mean and gray bar indicates HAI titer of 40 corresponding to 50% protective titer. Statistical significance was determined using Kruskal–Wallis test for multiple comparisons **P < 0.01. (*B*) Fold change of HAI titer (postvaccine HAI/prevaccine HAI) in Indigenous and non-Indigenous Australians. Red bar indicates mean and above the gray background indicates seroconversion (fourfold titer rise). (*C*) Effect of vaccination on frequency of 50% protective titers in Indigenous and non-Indigenous donors. Yellow and purple pie parts indicate donors that had HAI titers below 40 after vaccination. H3N2 includes 2018 donors and H1N1 includes 2018 and 2017 cohorts due to changes in the vaccine components. IBV components were not changed during study period and includes cohorts from all three years (*B* and *C*). (*D*) Breadth of antibody responses against previously circulating viruses in the 2018 cohort. Lines indicating geometric mean with 95% confidence interval plots were generated using geom_smooth function in R with loess. Stars indicate significant differences between Indigenous and non-Indigenous participants prior to (black) and after (red) vaccination calculated with Mann–Whitney *U* test (*P < 0.05, **P < 0.01). (*E*) PCA plots with 95% probability ellipse encompassing all HAI titers prior to vaccine (*Left*) and after vaccination (*Right*).

Based on the World Health Organization (WHO) guidelines, the best-defined correlate of protection against influenza virus infection is the hemagglutinin inhibition titer (HAI), which measures serum antibodies that prevent influenza virus–mediated agglutination of red blood cells. An HAI titer of 40 [log_2_(HAI/10) = 2] correlates with 50% protection in a healthy adult population (21). Significant increases in HA-specific antibodies were observed against all vaccine components following influenza vaccination in both Indigenous and non-Indigenous LIFT-V groups (Fig. 2*A*), with comparable fold-change rises in antibody titers observed in both groups (Fig. 2*B*). Notably, the response to the H3N2 Sing/2016 component was relatively poor and on average just reached the 50% protection threshold postvaccination [mean log2(HAI/10) = 2.13 in Indigenous vs. 2.33 in non-Indigenous] (Fig. 2*A*). This went in hand with a reduced titer changes postvaccination (geometric mean of fold change 1.69 to 1.85 vs. 2.48 to 3.79 of other vaccine components) (Fig. 2*B*). As a result, fewer individuals had protective titers against H3N2 compared with all the other vaccine components (Fig. 2*C*). Although 27 to 28% of individuals had postvaccine HAI titers below 40 for H3N2, our data showed that H3N2 titers increased from titers <40 to ≥40 postvaccination for 28 to 33% of individuals, indicating that vaccination was beneficial toward H3N2 for at least a third of individuals. For all other vaccine components, only a small proportion of individuals had HAI titers <40 postvaccination (5 to 9%), with 33 to 60% of individuals having pre-HAI titers of ≥40 perhaps due to prior vaccinations or infections. Nevertheless, in a substantial subset of individuals (20 to 37%) who had prevaccine titers <40, we observed an increase after vaccination ≥40, indicating the benefits of the QIV to promote antibody responses to protect individuals from influenza virus infection.

It is well known that older individuals develop lower titers after QIV (22), and most research has focused on improving vaccine responses in these high-risk groups. When we analyzed the postvaccine titers against age (*SI Appendix*, Fig. S1*A*) we observed a weak to moderate negative correlation in the non-Indigenous cohort to all vaccine components except for B/Vic/Bris/2008 (r_s_ between −0.34 and −0.57). Surprisingly, we did not observe any age-related negative correlations in our Indigenous cohort (*SI Appendix*, Fig. S1*A*).

### Back-Boosting of Antibodies Specific for the Previously Circulating Influenza Virus Strains.

Influenza virus infection and vaccine history can shape the breadth and magnitude of antibody responses directed toward the vaccine strain but also toward previous circulating strains (23). To analyze whether the breadth of the antibody repertoire differed between Indigenous and non-Indigenous donors, HAI titers were determined against influenza strains circulating between 1934 (A/Puerto Rico/8/1934) and 2016 (A/SINGAPORE/INFIMH-16-0019/2016) (*SI Appendix*, Table S2). For comparison, HAI landscapes were drawn for the 2018 cohort, which included the most participants (Fig. 2*D*). Overall, the magnitude and breadth of the antibody response were comparable between Indigenous and non-Indigenous participants (Fig. 2*D*). However, Indigenous Australians had significantly lower prevaccine titers against A/H3N2/Panama/1999 (PN/1999), B/Massachusetts/2012 (MAS/2012), and B/Sydney/2014 (SYD/2014) strains when compared with non-Indigenous donors and significantly lower titers against A/H1N1/Fukushima/2006 (FUK/2006), PN/1999, A/H3N2/Brisbane/2007 (BRIS/2007), and all B/Yamagata strains (MAS/2012, PHU/2013, and SYD/2014) postvaccination. Although significant, the difference in HAI titers against previous H1N1 and H3N2 strains which circulated ∼15 to 20 y ago were minimal (1.3- to 2.1-fold) between Indigenous and non-Indigenous. These differences were greater for B/Yamagata strains where non-Indigenous donors had higher HAI titers against B/Yamagata strains from the past decade (Fig. 2*D* and *SI Appendix*, Fig. S2*A*).

Interestingly, 6 participants (3/59 Indigenous and 3/84 non-Indigenous) seroconverted to A/H1N1/PR8/1934 following QIV vaccination, suggesting a back-boosting of antibody responses to previous strains. Five out of the seroconverters were aged between 45 and 52 y, with one 23-y-old suggesting some degree of imprinting of first virus infection on antibody response. Only one of these donors did, however, seroconvert to the A/Brazil/11/1978 strain, which would be potentially closer related to the virus they first encountered. Importantly, this did not impact their ability to seroconvert to the A/H1N1/Michigan/2015 vaccine strain (*SI Appendix*, Fig. S2*B*). Vaccine-induced back-boosting toward previously circulating H3N2 virus strains was also observed with 2 individuals seroconverting to PN/1999 and 24 to the more recent BRIS/2007 strain. Interestingly, BRIS/2007 seroconverters had significantly higher titers to the A/H3N2/Singapore/2016 vaccine strain compared with nonseroconverters (*SI Appendix*, Fig. S2*B*). There was also a strong positive correlation between BRIS/2007 and Singapore/2016 titers (*SI Appendix*, Fig. S2*B*), which may be due to the cross-reactive nature of these antibodies. In fact, antibody titers significantly correlated with each other for all H3 viruses tested from BRIS/2007 onward, potentially due to the antigenic similarity of these viruses compared with the older virus tested (*SI Appendix*, Fig. S3). Finally, a principal component analysis (PCA) compiling all the strain titers at pre- and postvaccine time points showed that the cohorts were highly variable among individuals, with both Indigenous and non-Indigenous datapoints highly overlapping, indicating no overall differences in antibody responses pre- and postvaccination between each group (Fig. 2*E*). Therefore, we show robust antibody responses elicited by influenza vaccination in the high-risk Indigenous Australians population are comparable to those in non-Indigenous Australians.

### Influenza Vaccination Elicits Comparable cT_FH_1 Cell Activation.

**Fig. 3.**
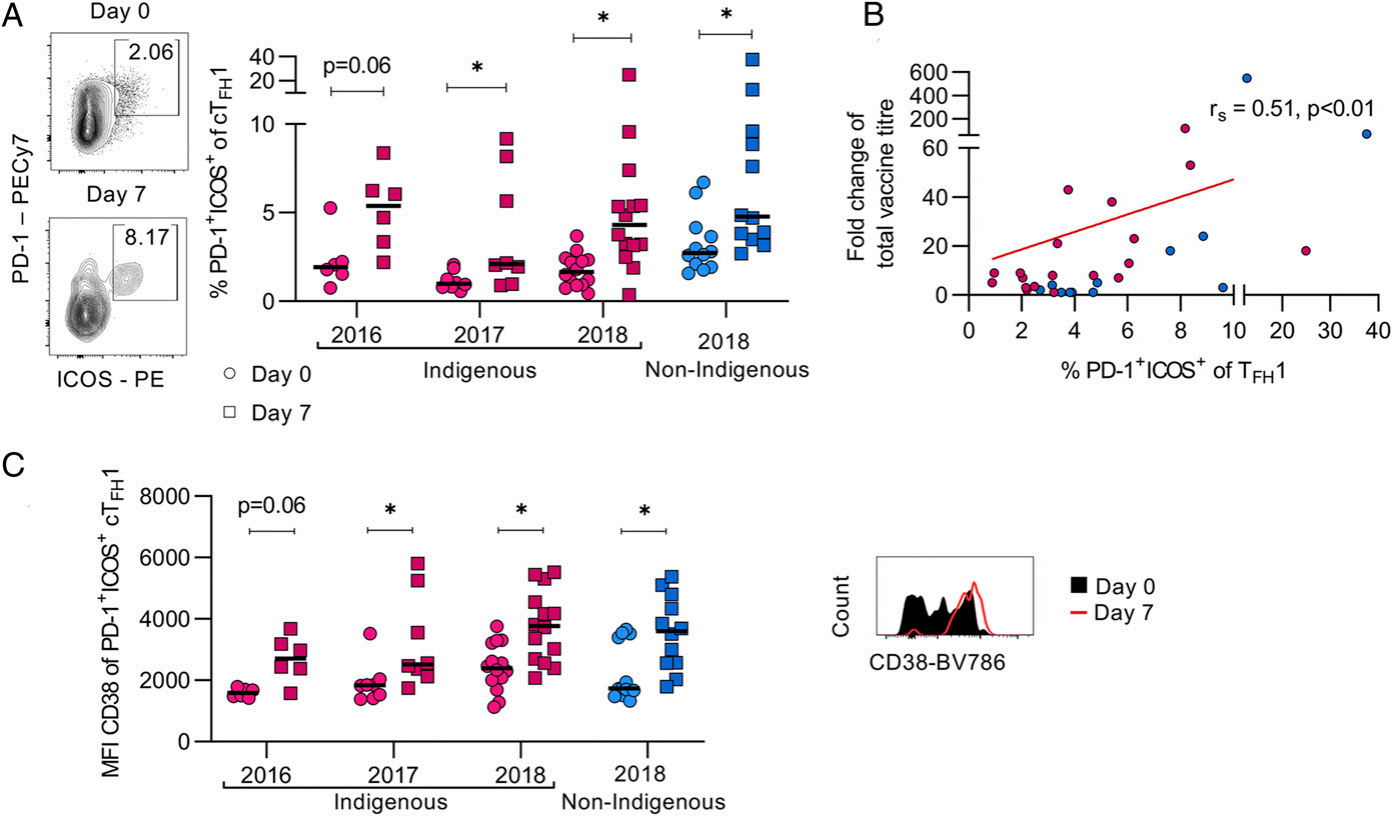
Vaccination induces activation of CD4^+^CXCR5^+^CXCR3^+^ cTfh cells in Indigenous and non-Indigenous donors. (*A*) Representative FACS plots and frequencies of PD1^+^ICOS^+^ cTfh1 cells postvaccination (day 7, squares) compared with baseline activation (day 0, circles). (*B*) Correlation (Spearman’s test) of titer fold titer change (d28/d0) and frequency of activated (PD1^+^ICOS^+^) cTfh1 cells 7 d postvaccination. (*C*) Geometric MFI of CD38 on activated (PD1^+^ICOS^+^) cTfh1 cells. (*Right*) A representative histogram of fluorescence intensity of CD38 on PD1^+^ICOS^+^ cTfh1 cells. Statistical significance was calculated with Mann–Whitney *U* test (*P < 0.05).

In previous studies, we described an increase in activated (PD1^+^ICOS^+^) cT_FH_1 cells (CD4^+^CXCR3^+^CXCR5^+^) at 7 d postvaccination, and these correlated with the rise in ASCs and HAI titers, indicating a robust vaccine response (9). Comparable levels of activated cT_FH_1 cells increased postvaccination in both Indigenous and non-Indigenous donors (Fig. 3*A*). Activation of cT_FH_1 cells also correlated moderately with the total fold change in titers (after/before) toward all the vaccine components (r_s_ = 0.51, *P* < 0.01) (Fig. 3*B*). Concomitantly, expression of the activation marker CD38 was also increased on activated cT_FH_1 cells postvaccination in both Indigenous and non-Indigenous donors, indicating highly activated cT_FH_1 cells in response to vaccination (Fig. 3*C*). However, vaccination did not induce expansion of cT_FH_17 (CD4^+^CCR6^+^CXCR3^−^CXCR5^+^) responses (*SI Appendix*, Fig. S4), in line with our previous findings (9).

### Activated Memory Influenza-Specific B Cells Increase after Vaccination.

**Fig. 4.**
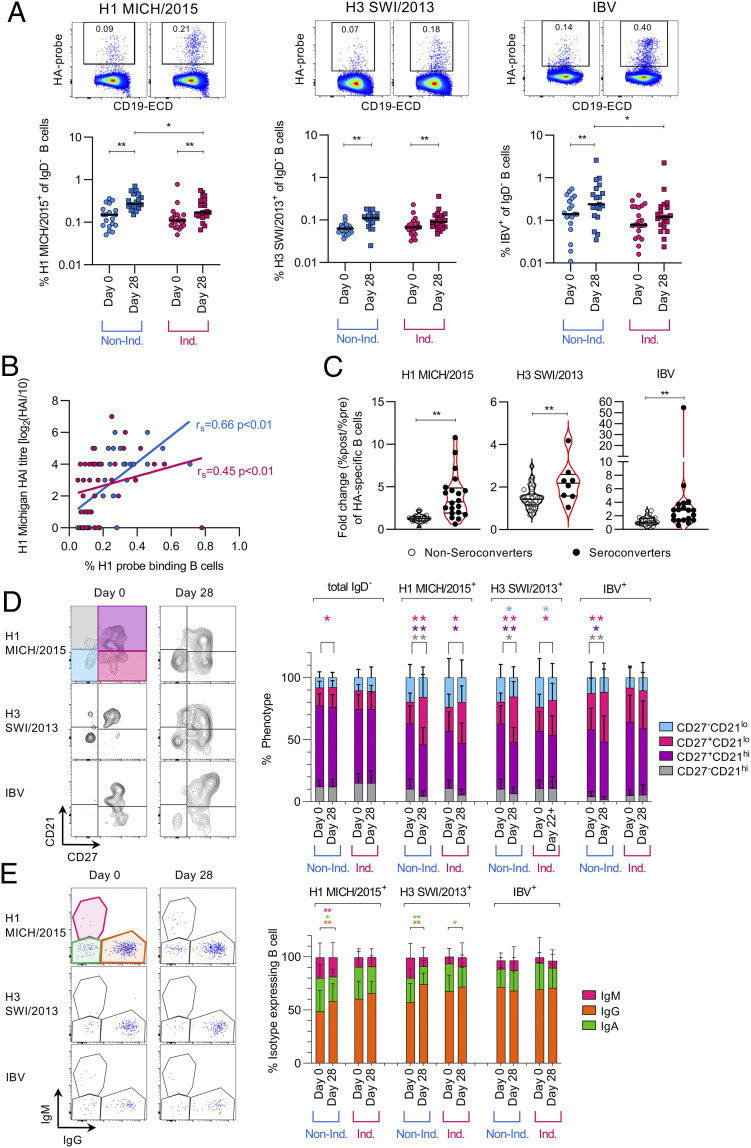
Higher frequencies of H1- and IBV-specific B cells postvaccination in non-Indigenous Australians. (*A*) Recombinant HA-specific IgD^−^ B cells were detected with fluorescently labeled recombinant HA proteins utilizing flow cytometry from the 2018 cohort. Statistical significance was calculated within the group using Wilcoxon matched-pairs signed rank test and differences between cohorts with Mann–Whitney *U* test (*P < 0.05, **P < 0.01). (*B*) Correlation (Spearman’s test) of the frequency of H1 Mich15-probe-specific B cells and H1 Mich15 antibody HAI titers. (*C*) Fold change in HA-specific B cells for each probe in seroconverters (fourfold or greater HAI titer change) and nonseroconverters. Statistical differences were calculated using Mann–Whitney *U* test. (*D*) Proportion of CD21/CD27 phenotype on HA-specific B cells and total IgD^−^ B cells. (*E*) Isotype expression of probe-specific cells and total IgD^−^ B cells. (*D* and *E*) Statistical differences were calculated using Wilcoxon-matched-pairs signed rank test with *P < 0.05 and **P < 0.01.

Formation and expansion of HA-specific B cells can be detected using well-established recombinant HA probes (9, 24). These probes consist of tetramerized HA molecules that have a removed transmembrane domain and a mutation (Y98F for IAV) to prevent nonspecific binding of the HA to B cells. As the probe for the A/H3N2/Singapore/2016 vaccine strain could not be successfully refolded after multiple attempts, we used an alternative A/Swi/2013 probe (2015 vaccine strain) previously used by our group which shows highly specific binding (9). H1-specific B cells were detected using a probe matched to the vaccine strain (A/Mich/2015) and probes to detect IBV-specific B cells were combined on the same fluorochrome (B/Bris/2008 and B/Phuket/2013 matching the two vaccine strains). HA-specific B cells detected across all the probes were assessed for donors from the 2018 vaccine cohort and were significantly increased postvaccination in Indigenous and non-Indigenous donors, except for IBV in Indigenous donors (Fig. 4*A*). The median frequency for all HA-specific B cells was between 0.06 and 0.27% of total CD19^+^IgD^−^ B cells. Interestingly, there was a trend for a lower frequency of HA-specific B cells in Indigenous donors compared with non-Indigenous donors at both pre- and postvaccination, which was significantly lower for H1N1 and IBV probes postvaccination (Fig. 4*A*), suggesting that Indigenous donors may have a lower memory precursor frequency of H1- and IBV-specific B cells. The frequency of H1-specific B cells moderately correlated (r_s_ = 0.58, *P* < 0.01) with A/H1N1/Mich/2015 HAI titers (Fig. 4*B*). For the H3 probe, there was a weak correlation with H3 titers (r_s_ = 0.27, *P* = 0.01). More importantly, seroconverters per vaccine strain had a higher fold-change increase (after/before percents) in their respective HA-specific B cells compared with nonseroconverters following vaccination (Fig. 4*C*).

Activation (CD21) and memory (CD27) markers were then analyzed on HA-specific B cells pre- and postvaccination. For all populations, with the exception of IBV in Indigenous donors, we detected a significant increase in the frequency of CD27^+^CD21^lo^ activated HA-specific B cells following vaccination (Fig. 4*D*), in line with our previous study in non-Indigenous donors only (9). These CD27^+^CD21^lo^ activated HA-specific B cells are hypothesized to be potential progenitors of long-lived plasma cells (25). An increase therefore might indicate the formation of lasting memory postvaccination in both Indigenous and non-Indigenous donors. However, the proportion of resting memory CD27^+^CD21^hi^ HA-specific B cells significantly decreased for all probes in the non-Indigenous donors and for the H1 probe in Indigenous donors (Fig. 4*D*). In terms of the isotype distribution, we detected a small but significant increase in proportion of immunoglobulin (Ig)G^+^ B cells in the H1- and H3-specific B cells, at the expense of IgA and IgM (H1 only), for non-Indigenous donors; there were no changes to the IgG distribution in Indigenous donors following vaccination (Fig. 4*E*). IgA expression was determined as (CD19^+^IgD^−^IgM^−^IgG^−^) as validated previously (9). This supports our previous study where vaccination did not induce marked changes to the isotype distribution of influenza-specific B cells (9) but is in contrast to influenza virus infection where both IgG^+^ and IgA^+^ HA-specific B cells were prominent during acute infection, before becoming predominantly IgG^+^ at the convalescent phase (26). Taken together, vaccination induced the expansion of activated memory HA-specific B cells which were predominantly IgG^+^ in both Indigenous and non-Indigenous donors.

### In-Depth Antibody Characterization Reveals Differences in Antibody Isotypes between Indigenous and Non-Indigenous Donors.

To complement the gold-standard HAI assay, which targets antibodies that can block receptor binding of the virus, we used Luminex multiplex technology and antibody characterization to generate a more in-depth analysis of influenza-specific serum antibodies that target a total of 10 different HA, NA, and NP proteins from various IAV and IBV strains to assess their isotype distribution (IgG1, IgG2, IgG3, IgG4, IgA1, IgA2, and IgM) and potential to engage with Fcγ receptors (FcγRIIa and FcγRIIIa) following influenza vaccination. Studies examining humoral immunity against influenza viruses suggest that specificity, isotype, and subclass can contribute to a range of potentially protective functions (27). To address this, microbeads were coated with recombinantly expressed influenza proteins for complete HA (A/Sing/2016, A/Swi/2013, A/Mich/2015, A/Cal/2009, and B/Phu/2013), HA1 subunit (A/HK/2014), HA_stem_ (A/Cal/2009), NA (A/HK/2014 and A/Cal/2009), and NP (A/Cal/2009), incubated with plasma samples, and analyzed with a variety of detectors (αIgG1, αIgG2, αIgG3, αIgG4, αIgA1, αIgA2, αIgM, dimeric recombinant soluble FcγRIIa, and dimeric rsFcγRIIIa). The magnitude of the antibody response/FcγR engagement for each influenza antigen (analyte) was measured by the median fluorescence intensity (MFI) of all recorded beads per sample pre- and postvaccination. FcγR engagement is responsible for antibody-dependent cellular cytotoxicity and antibody-dependent cellular phagocytosis, which is crucial to combat influenza virus infections (28).

**Fig. 5.**
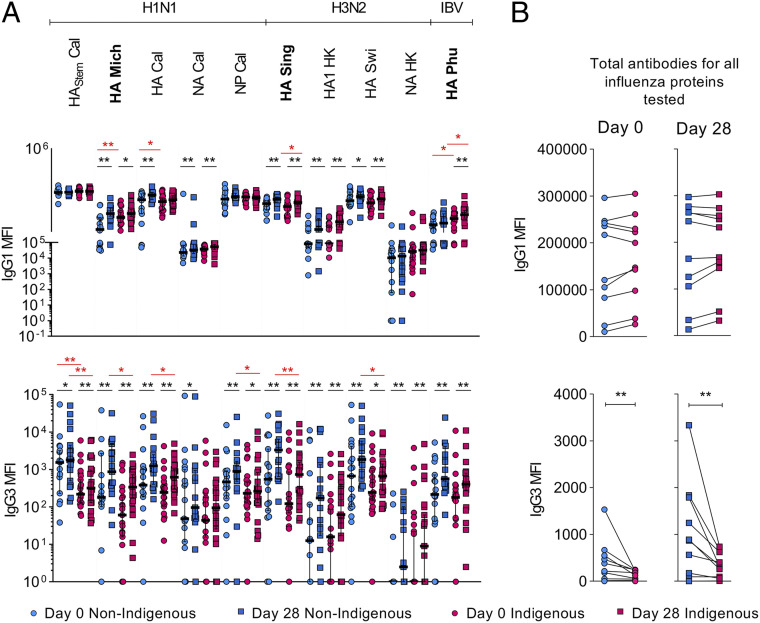
Influenza protein multiplex reveals differential patterns of influenza-specific IgG isotypes between Indigenous and non-Indigenous Australians. (*A*) Antibodies binding to influenza protein–coated fluorescent beads were detected with a secondary fluorescently conjugated detection antibody (for IgG1 and IgG3) in a multiplex bead array. MFI was determined per sample before and after (d28) vaccination. Bars indicate median, black stars (*P < 0.05, **P < 0.01) depict significance within each cohort, and red stars indicate statistical difference between Indigenous and non-Indigenous cohorts. Statistical significance was determined within each group with a Wilcoxon matched-pairs signed rank test and between cohorts using Mann–Whitney *U* test. (*B*) For each analyte in each detector, the median was calculated and compared in a paired analysis between Indigenous and non-Indigenous donors (e.g., median prevaccination HA Sing Indigenous vs. median prevaccination HA Sing non-Indigenous) to detect differences for each detector. The same analyte between Indigenous and non-Indigenous cohort is connected by a line. Statistical significance was determined using a Wilcoxon matched-pairs signed rank test.

Comparing pre- and postvaccination antibody levels in a subset of the 2018 vaccine cohort, we detected high increases for IgG1, IgG3, IgA1, FcγRIIa, and FcγRIIIa MFIs toward almost all influenza antigens postvaccination (Fig. 5*A* and *SI Appendix*, Fig. S5*A*). Interestingly, the magnitude of responses toward the various influenza antigens differed across the detectors. For IgG1, the highest median fold change was detected toward the HA1 A/HK/2014 protein (1.50), whereas the highest MFI fold change for IgG3 was toward HA A/Mich/2015 (4.12). This might indicate that different vaccine components induced different subclass responses, potentially due to different exposure history or glycosylation patterns. Unsurprisingly, we found only minor changes between pre- and postvaccine MFIs for IgG2, IgG4, IgA2, and IgM (*SI Appendix*, Fig. S5*A*), supporting a previous 2011 to 2012 vaccine study (29).

A significant increase in IgG1 antibodies was detected for H3 A/Sing, H3 (HA1) A/HK, H3 A/Swi13, H1 A/Mich, and N1 A/Cal for Indigenous and non-Indigenous donors postvaccination. While there was a significant increase in MFI for HA B/Phu detected in Indigenous donors, the opposite was observed for HA Cal, which was only significant in non-Indigenous donors. For H1 A/Mich, H1 A/Cal, and HA B/Phu significantly higher IgG1 levels were detected in Indigenous Australians prevaccination, with H3 A/Sing and HA B/Phu having significantly increased levels postvaccination (Fig. 5*A*).

The differences in IgG3 were much more pronounced compared with all other detectors with a significant increase for all antigens postvaccination, except for NA Cal in the Indigenous cohort. High antibody MFI changes were detected across all vaccine HA components (H3 A/Sing, H1 A/Mich, and HA B/Phu MFI fold change of 2.65, 4.12, and 2.73, respectively) as well as closely related components [H3 (HA1) A/HK and H1 A/Cal MFI fold change of 3.71 and 2.61, respectively]. IgG3 responses toward N2 A/HK and N1 A/Cal were lowest with MFI fold changes of 1.15 and 1.23, respectively. It was interesting to detect a significant increase in antibodies against the NP A/Cal (1.45 MFI fold change) as this is generally now assessed for vaccine responses. While the QIV was not depleted for NP, current vaccines are only quantified for HA and NA and might not harness the full potential of NP-specific antibodies that are reported to be protective in mice (30). Although we observed postvaccination increases in IgG3 responses for Indigenous and non-Indigenous donors, substantial differences in magnitude were observed between Indigenous and non-Indigenous donors across many of the analytes (Fig. 5*A*). Significant increases were also detected for engagement with FcγRIIa and FcγRIIIa using soluble dimers across all analytes (*SI Appendix*, Fig. S5*A*), which strongly correlated with IgG1 antibody responses (*SI Appendix*, Fig. S5*B*).

Since we observed differences for specific analytes between Indigenous and non-Indigenous donors, we compared the total detector-specific responses of Indigenous versus non-Indigenous donors at pre- and postvaccination. For this, we calculated the median MFI for each analyte and performed a Wilcoxon-matched-pairs signed rank test across all analytes. We found a strong bias for lower IgG3 responses in Indigenous donors compared with non-Indigenous pre- and postvaccination (Fig. 5*B*).

### Significant Differences in IgG3 Allotype Correlate with Lower Antibody Levels.

**Fig. 6.**
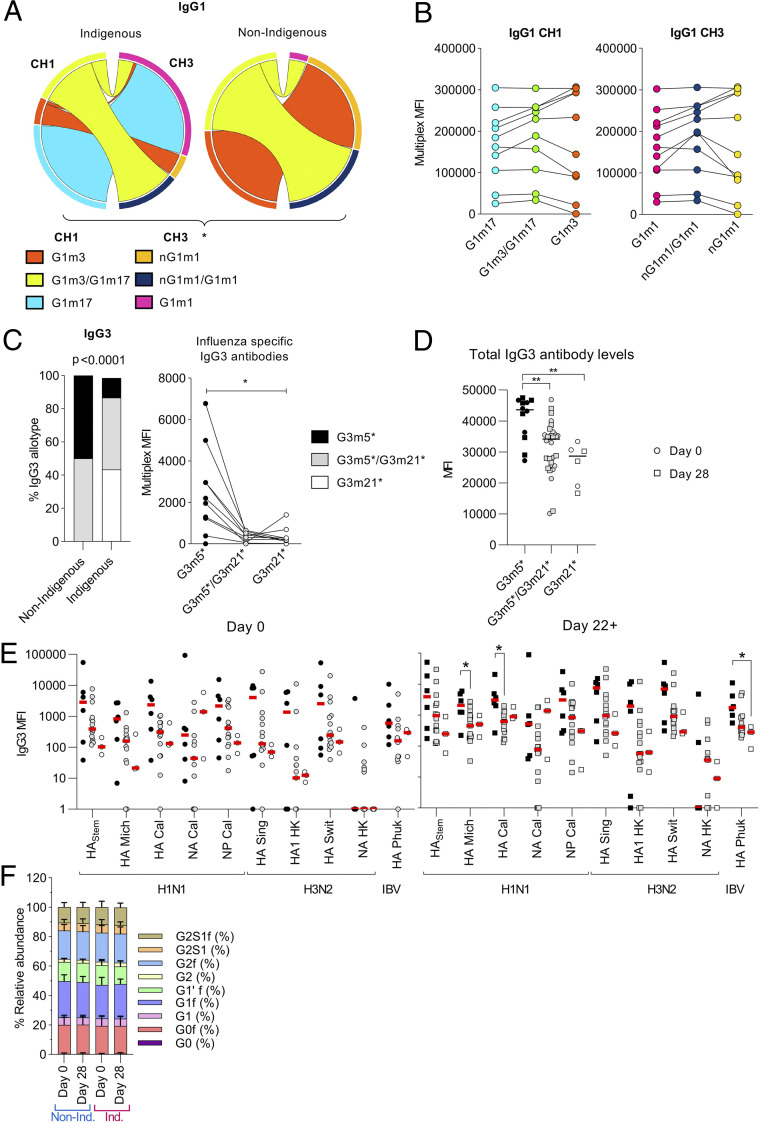
Higher IgG3 levels can be attributed to the G3m5* allotype. (*A*) Genomic DNA was sequenced for CH1 and CH3 allotypes (Indigenous *n* = 68, non-Indigenous *n* = 12). (*B*) Where available, MFIs were compared in paired analysis of each analyte between donors of different IgG1 CH1 allotypes (G1m17 *n* = 4 Individuals/8 datapoints, G1m3 *n* = 6 Individuals/12 datapoints, G1m17/G1m3 *n* = 14 Individuals/28 datapoints) and CH3 allotypes (G1m1 *n* = 6 Individuals/12 datapoints, nG1m1 *n* = 6 Individuals/ 12 datapoints, G1m1/nG1m1 *n* = 12 Individuals/24 datapoints). (*C*) Genomic DNA was sequenced for IgG3 CH2+3 allotypes (Indigenous *n* = 67, non-Indigenous *n* = 12). Median of each analyte was compared in paired analysis (G3m21* *n* = 3 Individuals/6 datapoints, G3m5* *n* = 6 individuals/12 datapoints, G3m21*/G3m5* n =15 Individuals/30 datapoints). (*D*) Total IgG3 levels were determined in multiplex using anti-IgG conjugated beads with anti-IgG3 fluorescent conjugated detection antibody. (*E*) IgG3 levels for the different analytes were plotted to compare antigen-specific IgG3 levels between different allotypes. (*F*) Total IgG glycosylation was mapped using LabChip GXII Microchip-CE electrophoresis system. Glycans were analyzed before and after (d28) vaccination for Indigenous (*n* = 12) and non-Indigenous (*n* = 9) donors. (*A* and *C*) Different distribution of IgG allotypes were calculated using Fisher’s exact test. (*D* and *E*) Statistical differences were determined using Kruskal–Wallis test (*P < 0.05, **P < 0.01).

To understand why some IgG1 and IgG3 isotype responses were lower in Indigenous donors we investigated their allotype distribution. IgG allotypes refer to polymorphisms in the constant region of the antibody and can affect its longevity in plasma as well as FcγR binding (31) or concentration (32). Analysis of the IgG1 allotypes revealed a significant difference in with higher frequencies of the G1m17 (65%) in the CH1 region and G1m1 allotypes (90%) in the CH3 region of Indigenous donors, while G1m3 (100%) in the CH1 region and nG1m1 alleles (91%) in the CH3 locus were predominantly found in non-Indigenous donors (Fig. 6*A*). However, when all the donors were grouped per IgG1 allotype for each locus we could not detect any significant differences in the multiplex MFI levels across all analyte (Fig. 6*B*). Analysis of IgG3 allotypes also detected significant differences between Indigenous and non-Indigenous Australians (higher G3m21* expression in Indigenous [88%] and 100% G3m5* expression in non-Indigenous Australians) (Fig. 6*C*). Interestingly, the expression of homozygous G3m5* significantly correlated with higher antibody responses per MFIs for each analyte (Fig. 6*C*), as well as the total MFI response (sum of individual MFIs per analyte) (Fig. 6*D*). More specifically, postvaccine responses toward H1 A/Mich, H1 A/Cal, and HA B/Phu were significantly higher in G3m5* homozygote individuals (Fig. 6*E*). These findings based on allelic expression are in line with the lower IgG3 responses found in Indigenous Australians and support a previous report of lower concentrations of IgG3 in G3m21* homozygous individuals (33).

### Influenza Vaccination Does Not Induce Changes in IgG Glycosylation Patterns.

Glycosylation is a posttranslational modification of antibodies by adding carbohydrate groups to the asparagine residues of IgG antibodies which alters the interaction with Fc receptors and mediated functions. Differences in glycosylation patterns have been observed between different populations around the world (34). However, no differences in glycosylation patterns were observed between a subset of Indigenous and non-Indigenous donors (Fig. 6*F*). Although previous influenza vaccine studies could not detect a difference in total IgG glycosylation, different glycosylation patterns have been observed at baseline between seroconverters and nonseroconverters (35), which has been proposed to serve as a biomarker to predict vaccine responses prior to vaccination. However, we did not observe any differences between pre- and postvaccination (Fig. 6*F*).

## Discussion

Annual vaccination is to date the only protection against seasonal influenza virus infections. Given that influenza vaccination is recommended for every individual >6 mo old, it is important to understand the immune responses induced by vaccination as additional measures of vaccine efficacy, especially in specific high-risk groups, including Indigenous Australians. Previously, we comprehensively defined optimal immune responses induced by TIV/QIV in non-Indigenous Australians (9). Studies in Aboriginal Canadians revealed antibody responses after vaccination with an AS03-adjuvanted pandemic pH1N1 vaccination comparable to non-Indigenous Canadians (19). While these data are encouraging for influenza virus vaccinations it was not known how these data translate to responses to the seasonal nonadjuvanted vaccine and if the vaccine induces robust memory formation. In the current study we defined humoral and cellular immune responses induced by the QIV in the high-risk Indigenous Australian population and coupled these analyses with in-depth antibody characterization and host genetic information. Our study provides evidence that Indigenous Australians mount effective, broad, and prototypical immune responses to the inactivated influenza vaccine. This includes antibodies toward the vaccine strains as well as back-boosting of antibodies specific for previously circulating strains, cT_FH_1 cells, and memory influenza-specific B cell populations, all at the levels found in non-Indigenous individuals.

We found increased serum antibody levels after vaccination for all vaccine components with the lowest postvaccine HAI titers detected toward the 2018 H3N2 component (Sing/2016). In contrast to the 2018 H3N2 response, we detected high titers to the 2016/17 component, indicating that antibody responses are not inherently reduced toward H3N2 viruses. Previous studies found, however, that antibodies against the 2016 vaccine component did not predict protection against infection potentially due to mutations that occur during egg passage of the vaccine component (36). Future studies are, however, needed to follow up vaccinated patients to understand whether the antibody levels provide comparable protective immunity against influenza disease in Indigenous Australians and non-Indigenous Australians.

Broad back-boost of antibodies was detected for H1N1, which even included for six participants the A/PR8 strain that circulated 84 y ago. A/PR8 and the pH1N1 share the same origin but differ in their antigenic sites, which might explain the back-boosting observed in some participants. However, titers to prepandemic H1N1 viruses correlated very weakly with titers to postpandemic H1N1 viruses. Therefore, these cross-reactive antibodies seem to have minimal impact on responses to the vaccine, given that we could not detect differences in the vaccine titer in individuals that seroconverted to A/PR8 in comparison with individuals who had no detectable titer changes to A/PR8. A different pattern was observed for H3N2 viruses, which circulated since 1968 in the human population. We identified back-boosting to previously circulating strains, as previously described (23). A strong correlation of all titers toward all H3N2 viruses, except for A/Port Chalmers/1/1973, indicates cross-reactivity between the antibodies, which is interesting given the general lower vaccine effectiveness of the H3N2 component.

Activated cT_FH_1 responses were significantly increased on d7 postvaccination in the Indigenous population across all the years. Significantly higher frequencies of memory HA-specific B cells against H1 and H3 vaccine components were also observed in our Indigenous and non-Indigenous donors. HAI titers against H1 Michigan positively correlated with the frequency of HA-H1–specific B cells. Moreover, seroconverters for H1, H3, or IBV had significantly higher fold changes in HA-specific B cells (percent after/percent before) compared with nonserocoverters. Postvaccination, these memory HA-specific B cells increased in activated memory phenotype and were predominantly IgG^+^, reminiscent of prototypical vaccine-induced immune responses in our previous study (9) but in contrast to acute influenza virus infection which comprises both IgG^+^ and IgA^+^ HA-specific B cells before becoming IgG^+^ at the recovery phase (26). Our data provide evidence that QIV induces activation and increases of influenza-specific B cells in Indigenous Australians, crucial for the formation of memory and the protective effect of vaccination.

Our in-depth antibody characterization approach indicated a reduced level of IgG3 in our Indigenous cohort, potentially linked to G3m21* allotype expression in line with a previous publication (33). The reason for this deficiency might be potentially derived from an upstream *cis* element that affects isotype switching (33). An IgG3 deficiency was associated during the 2009 influenza pandemic in a case report with high severity (37). This is highly interesting given the high severity and mortality during the 2009 pandemic in Indigenous Australians. Our results, however, only show a reduction in IgG3 levels, not necessarily a deficiency, as IgG3 levels did still increase even in the G3m21*^+/+^ individuals. Therefore, further experiments are needed to quantify IgG3 levels in a bigger cohort for conclusive data to indicate whether our findings might have physiological significance.

Our LIFT-V cohort displayed broad HLA-II profiles in Indigenous Australians, and these were comparable to the non-Indigenous individuals. We detected higher frequencies of DPB1*05:01, DQB*05:03, DQB*06:01, DRB1*08:03, and DRB1*14:08 in Indigenous Australians compared with non-Indigenous individuals. Given the comparable vaccine responses between the Indigenous and the non-Indigenous cohorts, it appears that the differences in the HLA-II profiles did not lead to altered vaccine responses.

One limitation of our study is the missing data of comorbidities in the cohort. Comorbidities including obesity and renal disease are increased in Indigenous Australians (13) and could affect vaccine responses. Further studies need to include these factors to assess the impact of these comorbidities in Indigenous Australians on vaccine responses and demonstrate that vaccination does also induce robust vaccine responses in individuals with underlining risk factors.

In conclusion, we identified robust adaptive immune responses in Indigenous Australians toward the inactivated influenza vaccine which were comparable to those observed in non-Indigenous Australians. This is of high importance, given the misgivings of Indigenous populations that the vaccine might not be protective (18). Our data provide immunological evidence of effective QIV responses being generated in Indigenous Australians. The results from our study advocate an immunological basis for the potential of influenza vaccination to protect Indigenous Australians from the high impact of seasonal influenza virus infections and their consequent complications, including cardiac disease such as myocardial infarction and endocarditis, long-term lung injury, and secondary bacterial infections.

## Methods

### Human Ethics.

All experiments were conducted according to the Declaration of Helsinki principles and NHMRC Code of practice and approved by the University of Melbourne Human Research Ethics Committee (#1955465 and #1441452), the Human Research Ethics Committee of the Northern Territory Department of Health and Menzies School of Health Research (#2012-1928), and the Tasmanian Human Research Ethics Committee (#H0015460). All participants provided informed consent.

Healthy individuals of Indigenous status were recruited in Darwin, located in the Northern Territory (NT), Australia. Individuals were vaccinated, and heparinized peripheral blood (peripheral blood mononuclear cells and plasma) and serum tubes were collected. Samples were taken on d0 (2016 *n* = 6, 2017 *n* = 10, 2018 *n* = 66), d7 (2016 *n* = 6, 2017 *n* = 8, 2018 *n* = 14), and d28 (2016 *n* = 6, 2017 *n* = 7, 2018 *n* = 46; median time after vaccination 30 d [22 to 84 d postvaccination]). Samples were processed at Menzies School of Health Research and stored at the University of Melbourne. Non-Indigenous participants were recruited in Darwin, NT (2018), Melbourne, VIC (2016, 2017, and 2018) and Launceston, TAS (2018). These samples were taken on d0 (2016 *n* = 29, 2017 *n* = 22, 2018 *n* = 33), d7 (2018 *n* = 12), and d28 (2016 *n* = 29, 2017 *n* = 22, 2018 *n* = 33; median time after vaccination 28 d [26 to 85 d postvaccination]). Three donors in the LIFT-V cohort participated in 2016 and 2017 and one donor participated in all three years (*SI Appendix*, Table S1). In cases where less than the total number of samples were analyzed (rHA probes, multiplex and glycosylation), subsets were matched for age and antibody responses between Indigenous and non-Indigenous donors as close as possible. Genotyping, HAI assay, surface staining of B and T cells IgG glycosylation, and multiplex assays are described in *SI Appendix*.

## Supplementary Material

Supplementary File

## Data Availability

All study data are included in the article and/or *SI Appendix*.
