# Supplementary material for: Robust and prototypical immune responses toward influenza vaccines in the high-risk group of Indigenous Australians
Source: Proc Natl Acad Sci U S A. 2021 Oct 4;118(41):e2109388118. doi: 10.1073/pnas.2109388118 (PMC8522271; doi:10.1073/pnas.2109388118)
Supplement: Supplementary File [file pnas.2109388118.sapp.pdf]

SUPPLEMENTARY INFORMATION TEXT

METHODS

HLA-II typing. HLA-II genotyping was performed on genomic DNA isolated from granulocytes by the Victorian Transplant and Immunogenetics Service (West Melbourne, VIC, Australia) for all donors who consented to genetic analysis**.**

Hemagglutinin Inhibition assay (HAI). HAI titration was performed as described previously(1) and per WHO guidelines(2) on serum. H3N2 viruses were titrated in the presences of Oseltamivir carboxylate on guinea pig red blood cells whereas IBV and H1N1 viruses were titrated without Oseltamivir carboxylate on turkey red blood cells. IBV viruses were ether treated before titration.

Surface staining of B and T cells. Frozen PBMCs were thawed in RPMI with Benzonase® Nuclease (MerkMillipore, MA, USA). Cells were stained with panel A (Table S3.) for early responses or panel B (Table S3.) to analyse HA-specific B cells and fixed in 1% PFA before acquiring. Samples were acquired on a LSR Fortessa (BD Biosciences) and analysed with FlowJo 10 (FlowJo, LLC).

IgG1 and IgG3 allotypes. IgG1 allotypes were analysed using genomic DNA isolated from granulocytes. A PCR with specific primers for either IgG1 CH1 (for: 5'-CCCCTGGCACCCTCCTCCAA-3'/ rev: 5'-GCCCTGGACTGGGGCTGCAT-3' (3)), CH3 (for: 5'-GAGCCCAAATCTTGTGACAA-3'/ rev: 5'-GGCGATGTCGCTGGGA-3' (4)) or IgG3 CH2+3 (for: 5'-GTCGGGTGCTGA-CACATCTG-3'/ rev: 5'-CTTGCCGGCYRTSGCACTCA-3' (5)) was performed and DNA sequenced by Sanger sequencing. A subset of donors consenting to genetic analysis (n=46) were analysed (IgG1 allotypes in CH1 and CH3; IgG3 alloypes in the CH2 and CH3 region).

Total IgG glycosylation profile. Total IgG glycosylation was analysed using capillary electrophoresis as described previously(6). In brief, total IgG was purified from plasma using Melon gel IgG purification resin according to the manufacturer's protocol (Thermo Fisher). N-

linked glycans on purified IgG was analysed using LabChip GXII Touch Microchip-CE platform per manufacturer's protocol (Perkin Elmer).

Coupling of magnetic carboxylated beads. A custom influenza multiplex assay was designed with H1N1 HA Cal09(11055-V08H), H1N1 NA Cal09(11058-V08B), H1N1 NP Cal09(40205-V08B), H3N2 HA1 HK(40555-V08H), H3N2 NA HK(40569-V07H), and H3N2 HA Switz(40497-V08B) (Sino Biological) and H1N1 HA Mich(IT-003-00105ΔTMp) and H3N2 HA Sing(IT-003-00434ΔTMp) (Immune Technology) antigens, as well as H1N1 HA stem and IBV/Phuket HA (produced in-house). Tetanus toxoid (Sigma) and anti-human Fd IgG (#MAB1304; Sigma-Aldrich), as well as BSA blocked beads were included as positive and negative controls, respectively. Antigens were covalently coupled to magnetic carboxylated beads (Bio Rad) at a ratio of 10 million beads to 100 μg of antigen using a two-step carbodiimide reaction, as previously described(7). Beads were washed and activated in 100 mM monobasic sodium phosphate, pH 6.2, followed by the addition of Sulfo-N-hydroxysulfosuccinimide and 1-Ethyl-3-(3-dimethylaminopropyl) carbodiimide (Thermo Fisher Scientific). The microspheres were incubated at room temperature (RT) for 30 minutes and then washed three times and resuspended in 50 mM MES pH 5.0 (Thermo Fisher Scientific). Respective antigens and activated beads were combined and rotated at RT in the dark for 3 hrs. Beads were washed with PBS and blocked for 30 minutes in blocking buffer (PBS, 0.1% BSA, 0.02% TWEEN-20, 0.05% Azide, pH 7), before being washed and resuspended to 1 million beads per 100 μL in PBS 0.05% Sodium Azide.

Luminex bead-based multiplex assay. A multiplex assay was used to assess the isotypes and subclasses of influenza-specific antibodies present in donor plasma, as described previously(7, 8). Briefly, 50 μL of working bead mixture containing 600 beads per bead region and 50 μL of diluted plasma were added per well to a black, clear bottom 96-well plate (Greiner Bio-One) and shaken overnight at 4ºC. The plate was washed with PBS containing 0.05% Tween20 (PBST) and influenza-specific-antibodies detected using phycoerythrin (PE)-conjugated mouse anti-human IgG1-4, IgA1-2, and IgM (#9052-09, #9070-09, #9210-09, #9200-09, #9130-09, #9140-09, #9020-09) (Southern Biotech), at 1.3 μg/mL, 50 μL per well. The detection antibody was shaken for 2 hrs at RT, the plate washed, and beads resuspended in 100 μL of sheath fluid. After shaking at RT for 10 minutes, the plate was read by the FlexMap 3D

(Luminex) and the binding of the PE-detector was measured to calculate the median fluorescence intensity (MFI). Double background subtraction was performed with the removal of the blank (buffer only) wells, as well as the BSA-blocked control bead background signal removed for each well. For the detection of FcγR, soluble recombinant FcγR dimers (FcγRIIa-H131 and FcγRIIIa-V158) were provided by Bruce Wines and Mark Hogarth. Dimers were added at 1 μg/mL, 25 μL per well, and the plate shaken at RT for 2 hrs and washed. Streptavidin, R-Phycoerythrin conjugate (SAPE; Invitrogen) was added at 1 μg/mL, 50 μL per well, shaken at RT for 2 hrs before being washed and read as mentioned above.

Statistical analysis. Significance was assessed using Wilcoxon matched-pairs signed-rank test (for changes from baseline) or Kruskal-Wallis test (for comparisons across groups and timepoints) adjudstments for multiple testing are indicated in the figure legends. Mann-Whitney test was used to compare unpaired samples, and a Wilcoxon-matched-pairs signed rank test was used to compare paired samples with graphpad prism v.9.0. Correlations were assessed using Spearman's correlation coefficient (r_s) for non-Gaussian distributions. As a visual guidance, linear correlation was drawn to indicate a correlation between assessed parameter. PCA was generated using R v.4.0.2 using *prcomp* function.

1. D. Hobson, R. L. Curry, A. S. Beare, A. Ward-Gardner, The role of serum haemagglutination-inhibiting antibody in protection against challenge infection with influenza A2 and B viruses. *J. Hyg. (Lond).* **70**, 767–777 (1972).
2. Who, Manual for the laboratory diagnosis and virological surveillance of influenza. *World Heal. Organ. 2011*, 153 (2011).
3. M. Balbin, A. Grubb, M. Abrahamson, R. Grubb, Determination of allotypes G1m(f) and G1m(z) at the genomic level by subclass-specific amplification of DNA and use of allele-specific probe. *Exp. Clin. Immunogenet.* **8**, 88–95 (1991).
4. C. I. Webster, *et al.*, A comparison of the ability of the human IgG1 allotypes G1m3 and G1m1,17 to stimulate T-cell responses from allotype matched and mismatched donors. *MAbs* **8**, 253–263 (2016).
5. M. Dambrun, *et al.*, Human immunoglobulin heavy gamma chain polymorphisms: Molecular confirmation of proteomic assessment. *Mol. Cell. Proteomics* **16**, 824–839 (2017).
6. A. E. Mahan, *et al.*, A method for high-throughput, sensitive analysis of IgG Fc and Fab glycosylation by capillary electrophoresis. *J. Immunol. Methods* **417**, 34–44 (2015).
7. K. Selva, *et al.*, Distinct systems serology features in children, elderly and COVID patients (2020) https:/doi.org/10.1101/2020.05.11.20098459.
8. E. P. Brown, *et al.*, High-throughput, multiplexed IgG subclassing of antigen-specific antibodies from clinical samples. *J. Immunol. Methods* **386**, 117–123 (2012).

Fig. S1. Negative correlation between HAI and age in non-Indigenous but not Indigenous Australians. Correlation (Spearman's test) of post vaccine HAI titres in Indigenous (pink) and non-Indigenous Australians (blue).

Fig. S2. Individual antibody titres detected by HAI. (**A**) Individual HAI titres that were measured to determine the breadth of antibody response as summarized in **Fig. 2**. (**B**) Post-vaccine titres to A/Michigan/2015 and A/Singapore/2016 in seroconverters to A/PR/1934 and A/Bris2007, respectively, and correlation (Spearman's test) of A/Bris2007 and A/Singapore2016 titres in Indigenous and non-Indigenous Australians. (**A, B**) Significance was calculated using Wilcoxon matched-pairs signed ranks test (*$p<0.05$; **$p<0.01$).

Fig. S3. Correlation of HAI titres. Correlation of HAI titres was calculated using non-parametric Spearman's correlation. Colours and size of circles indicate correlation coefficient value with a adjusted $p<0.05$ by False-discovery-rate adjustment in R.

Fig. S4. Early activation of cTfh17 cells post vaccination. Frequency of PD-1$^+$ICOS$^+$ cTfh17 (CD4$^+$CXCR5$^+$CCR6$^+$CXCR3$^-$) cells at baseline (day 0) and day 7 post vaccination in Indigenous and non-Indigenous donors. Significance was calculated using Wilcoxon matched-pairs signed ranks test (*$p<0.05$).

Fig. S5. Influenza protein multiplex for IgG2, IgG4, IgM, IgA1, IgA2 or fluorescently labelled Fcγ receptor dimers (FcγRIIa and FcγRIIIa). (**A**) Antibodies binding to influenza protein coated fluorescent beads were detected with a secondary fluorescently conjugated detection antibody in a multiplex bead array. Median fluorescence intensity was determined per sample prior and post (d28) vaccination. Bars indicate median, black stars (*$p<0.05$, **$p<0.01$) depict significance within each cohort, red stars indicate statistical differences between Indigenous and non-Indigenous cohort. Statistical significance was determined within each group with a Wilcoxon matched-pairs signed rank test and between cohorts using Mann-Whitney test. (**B**) Correlation (Spearman's test) of FcγRIIa and FcγRIIIa MFI with IgG1 MFI.

Fig. S6. Gating of ASC and cTfh cells. Single, live cells were gated for CD19$^+$ B and CD3$^+$ T cell markers. B cells were gated for antibody-secreting cells (CD38$^+$CD27$^+$) whereas T cells were further dissected on CD4, CXCR5, CXCR3 and CCR6 expression to characterise activation of cTfh cells (PD-1$^+$ICOS$^+$).

Fig. S7. Gating strategy for HA-specific B cells. Single B cells (CD19$^+$CD10$^-$CD14$^-$CD3$^-$CD8$^-$CD16$^-$FreeSA$^-$) were gated for IgD and frequency of probe-specific cells determined for IgD$^-$ B cells**.**

Table S1. Donor demographics of the QIV vaccine cohort.

Donor ID	Follow-up	Age	Gender	Ind. Status	Year	Location	Sample
LIFT-V 125	Yes	36	F	Indigenous	2016	Darwin	PBMC+serum
LIFT-V 126	Yes	59	F	Indigenous	2016	Darwin	PBMC+serum
LIFT-V 127	Yes	42	M	Indigenous	2016	Darwin	PBMC+serum
LIFT-V 128	Yes	54	M	Indigenous	2016	Darwin	PBMC+serum
LIFT-V 129	Yes	26	F	Indigenous	2016	Darwin	PBMC+serum
LIFT-V 130	Yes	31	F	Indigenous	2016	Darwin	PBMC+serum
LIFT-V 141	Yes	61	F	Indigenous	2017	Darwin	PBMC+serum
LIFT-V 142	Yes	32	F	Indigenous	2017	Darwin	PBMC+serum
LIFT-V 143	Yes	57	F	Indigenous	2017	Darwin	PBMC+serum
LIFT-V 144	No	27	F	Indigenous	2017	Darwin	PBMC+serum
LIFT-V 145	No	60	F	Indigenous	2017	Darwin	PBMC+serum
LIFT-V 146	Yes	60	F	Indigenous	2017	Darwin	PBMC+serum
LIFT-V 147/129	Yes	27	F	Indigenous	2017	Darwin	PBMC+serum
LIFT-V 148	Yes	32	F	Indigenous	2017	Darwin	PBMC+serum
LIFT-V 149	No	21	M	Indigenous	2017	Darwin	PBMC+serum
LIFT-V 153	Yes	43	M	Indigenous	2017	Darwin	PBMC+serum
LIFT-V 154	No	36	M	Indigenous	2018	Darwin	PBMC+serum
LIFT-V 155	Yes	32	M	Indigenous	2018	Darwin	PBMC+serum
LIFT-V 156	Yes	40	M	Indigenous	2018	Darwin	PBMC+serum
LIFT-V 157	Yes	59	F	Indigenous	2018	Darwin	PBMC+serum
LIFT-V 158	Yes	43	F	Indigenous	2018	Darwin	PBMC+serum
LIFT-V 159	Yes	44	M	Indigenous	2018	Darwin	PBMC+serum
LIFT-V 160	Yes	33	F	Indigenous	2018	Darwin	PBMC+serum
LIFT-V 161	Yes	56	F	Indigenous	2018	Darwin	PBMC+serum
LIFT-V 162	Yes	37	F	Indigenous	2018	Darwin	PBMC+serum
LIFT-V 163	Yes	56	F	Indigenous	2018	Darwin	PBMC+serum
LIFT-V 164	No	35	F	Indigenous	2018	Darwin	PBMC+serum
LIFT-V 165	Yes	64	F	Indigenous	2018	Darwin	PBMC+serum
LIFT-V 166	Yes	60	F	Indigenous	2018	Darwin	PBMC+serum
LIFT-V 167	Yes	46	F	Indigenous	2018	Darwin	PBMC+serum
LIFT-V 168	No	39	F	Indigenous	2018	Darwin	PBMC+serum
LIFT-V 169	Yes	51	F	Indigenous	2018	Darwin	PBMC+serum
LIFT-V 170	Yes	56	F	Indigenous	2018	Darwin	PBMC+serum
LIFT-V 171	No	31	F	Indigenous	2018	Darwin	PBMC+serum
LIFT-V 172	Yes	49	F	Indigenous	2018	Darwin	PBMC+serum
LIFT-V 173	Yes	61	F	Indigenous	2018	Darwin	PBMC+serum
LIFT-V 174	Yes	41	F	Indigenous	2018	Darwin	PBMC+serum
LIFT-V 175	Yes	48	F	Indigenous	2018	Darwin	PBMC+serum
LIFT-V 176	Yes	32	F	Indigenous	2018	Darwin	PBMC+serum
LIFT-V 177	Yes	31	F	Indigenous	2018	Darwin	PBMC+serum
LIFT-V 178	Yes	24	F	Indigenous	2018	Darwin	PBMC+serum
LIFT-V 179	Yes	34	F	Indigenous	2018	Darwin	PBMC+serum
LIFT-V 180	Yes	51	F	Indigenous	2018	Darwin	PBMC+serum
LIFT-V 181	Yes	28	F	Indigenous	2018	Darwin	PBMC+serum
LIFT-V 182	Yes	27	F	Indigenous	2018	Darwin	PBMC+serum
LIFT-V 183	Yes	59	F	Indigenous	2018	Darwin	PBMC+serum
LIFT-V 184	Yes	56	F	Indigenous	2018	Darwin	PBMC+serum
LIFT-V 185	Yes	44	F	Indigenous	2018	Darwin	PBMC+serum
LIFT-V 186	No	42	M	Indigenous	2018	Darwin	PBMC+serum
LIFT-V 187	No	43	F	Indigenous	2018	Darwin	PBMC+serum
LIFT-V 188	No	34	M	Indigenous	2018	Darwin	PBMC+serum
LIFT-V 189	No	55	M	Indigenous	2018	Darwin	PBMC+serum
LIFT-V 190	No	50	F	Indigenous	2018	Darwin	PBMC+serum
LIFT-V 191	No	45	F	Indigenous	2018	Darwin	PBMC+serum
LIFT-V 192	No	59	F	Indigenous	2018	Darwin	PBMC+serum
LIFT-V 193	Yes	41	F	Indigenous	2018	Darwin	PBMC+serum

LIFT-V 194	Yes	50	F	Indigenous	2018	Darwin	PBMC+serum
LIFT-V 195	Yes	62	F	Indigenous	2018	Darwin	PBMC+serum
LIFT-V 196	Yes	49	F	Indigenous	2018	Darwin	PBMC+serum
LIFT-V 197	No	46	F	Indigenous	2018	Darwin	PBMC+serum
LIFT-V 198	Yes	46	F	Indigenous	2018	Darwin	PBMC+serum
LIFT-V 199	No	36	F	Indigenous	2018	Darwin	PBMC+serum
LIFT-V 202	Yes	48	M	Indigenous	2018	Darwin	PBMC+serum
LIFT-V 203	Yes	39	F	Indigenous	2018	Darwin	PBMC+serum
LIFT-V 204	Yes	24	M	Indigenous	2018	Darwin	PBMC+serum
LIFT-V 205	Yes	45	F	Indigenous	2018	Darwin	PBMC+serum
LIFT-V 209	No	NA		Indigenous	2018	Darwin	PBMC+serum
LIFT-V 210	No	NA		Indigenous	2018	Darwin	PBMC+serum
LIFT-V 214	Yes	45	M	Indigenous	2018	Darwin	PBMC+serum
LIFT-V 215	No	32	F	Indigenous	2018	Darwin	PBMC+serum
LIFT-V 216	Yes	23	F	Indigenous	2018	Darwin	PBMC+serum
LIFT-V 217	Yes	27	F	Indigenous	2018	Darwin	PBMC+serum
LIFT-V 220	Yes	26	M	Indigenous	2018	Darwin	PBMC+serum
LIFT-V 221	No	23	M	Indigenous	2018	Darwin	PBMC+serum
LIFT-V 222	No	47	F	Indigenous	2018	Darwin	PBMC+serum
LIFT-V 223	Yes	49	F	Indigenous	2018	Darwin	PBMC+serum
LIFT-V 225	Yes	49	F	Indigenous	2018	Darwin	PBMC+serum
LIFT-V 226	Yes	65	F	Indigenous	2018	Darwin	PBMC+serum
LIFT-V 229	No	23	M	Indigenous	2018	Darwin	PBMC+serum
LIFT-V 230	Yes	27	F	Indigenous	2018	Darwin	PBMC+serum
LIFT-V 231	No	48	M	Indigenous	2018	Darwin	PBMC+serum
LIFT-V 232	No	24	F	Indigenous	2018	Darwin	PBMC+serum
LIFT-V 233	No	49	M	Indigenous	2018	Darwin	PBMC+serum
CJB	Yes	50	F	Non-Ind.	2016	Melbourne	Serum
DI	Yes	50	F	Non-Ind.	2016	Melbourne	Serum
KK 25	Yes	29	F	Non-Ind.	2016	Melbourne	Serum
KK 26	Yes	27	M	Non-Ind.	2016	Melbourne	Serum
KK 28	Yes	33	F	Non-Ind.	2016	Melbourne	Serum
KK 32	No	26	F	Non-Ind.	2016	Melbourne	Serum
KK 39	No	50	F	Non-Ind.	2016	Melbourne	Serum
KK 40	No	29	F	Non-Ind.	2016	Melbourne	Serum
KK 44	Yes	36	F	Non-Ind.	2016	Melbourne	Serum
KK 45	No	41	F	Non-Ind.	2016	Melbourne	Serum
KK 49	No	23	M	Non-Ind.	2016	Melbourne	Serum
KK 50	No	27	M	Non-Ind.	2016	Melbourne	Serum
KK 51	Yes	32	M	Non-Ind.	2016	Melbourne	Serum
KK 54	Yes	47	F	Non-Ind.	2016	Melbourne	Serum
KK 55	No	28	F	Non-Ind.	2016	Melbourne	Serum
KK 62	Yes	24	F	Non-Ind.	2016	Melbourne	Serum
KK 67	No	54	M	Non-Ind.	2016	Melbourne	Serum
KK 70	No	47	F	Non-Ind.	2016	Melbourne	Serum
KK 71	Yes	22	F	Non-Ind.	2016	Melbourne	Serum
KK 72	No	52	F	Non-Ind.	2016	Melbourne	Serum
KK 73	No	21	F	Non-Ind.	2016	Melbourne	Serum
KK 74	No	21	F	Non-Ind.	2016	Melbourne	Serum
KK 75	No	46	F	Non-Ind.	2016	Melbourne	Serum
KK 76	Yes	35	F	Non-Ind.	2016	Melbourne	Serum
KK 77	No	25	F	Non-Ind.	2016	Melbourne	Serum
KK 78	No	22	F	Non-Ind.	2016	Melbourne	Serum
KK 15	Yes	32	M	Non-Ind.	2016	Melbourne	Serum
KK 18	No	45	F	Non-Ind.	2016	Melbourne	Serum
KK34	No	28	M	Non-Ind.	2016	Melbourne	Serum
N1	Yes	40	M	Non-Ind.	2017	Melbourne	Serum
N3	Yes	28	F	Non-Ind.	2017	Melbourne	Serum
N4	Yes	34	F	Non-Ind.	2017	Melbourne	Serum
N5	Yes	34	F	Non-Ind.	2017	Melbourne	Serum

N6	Yes	28	F	Non-Ind.	2017	Melbourne	Serum
N7	Yes	44	F	Non-Ind.	2017	Melbourne	Serum
N8	Yes	24	F	Non-Ind.	2017	Melbourne	Serum
N9	Yes	25	M	Non-Ind.	2017	Melbourne	Serum
N10	Yes	31	M	Non-Ind.	2017	Melbourne	Serum
N11	Yes	30	F	Non-Ind.	2017	Melbourne	Serum
N12	Yes	26	M	Non-Ind.	2017	Melbourne	Serum
N13	Yes	55	M	Non-Ind.	2017	Melbourne	Serum
N14	Yes	40	F	Non-Ind.	2017	Melbourne	Serum
N15	Yes	50	F	Non-Ind.	2017	Melbourne	Serum
N16	Yes	34	F	Non-Ind.	2017	Melbourne	Serum
N17	Yes	29	M	Non-Ind.	2017	Melbourne	Serum
N18	Yes	30	F	Non-Ind.	2017	Melbourne	Serum
N19	Yes	32	F	Non-Ind.	2017	Melbourne	Serum
N20	Yes	28	M	Non-Ind.	2017	Melbourne	Serum
N21	Yes	27	M	Non-Ind.	2017	Melbourne	Serum
N22	Yes	29	M	Non-Ind.	2017	Melbourne	Serum
N23	Yes	31	F	Non-Ind.	2017	Melbourne	Serum
KK28	No	35	F	Non-Ind.	2018	Melbourne	PBMC+serum
KK42	Yes	36	M	Non-Ind.	2018	Melbourne	PBMC+serum
KK52	No	50	M	Non-Ind.	2018	Melbourne	PBMC+serum
KK94	No	52	M	Non-Ind.	2018	Melbourne	PBMC+serum
KK95	No	40	M	Non-Ind.	2018	Melbourne	PBMC+serum
KK96	Yes	31	F	Non-Ind.	2018	Melbourne	PBMC+serum
KK97	No	28	M	Non-Ind.	2018	Melbourne	PBMC+serum
KK98	No	29	M	Non-Ind.	2018	Melbourne	PBMC+serum
LIFT-V 200	Yes	40	F	Non-Ind.	2018	Darwin	PBMC+serum
LIFT-V 201	Yes	54	M	Non-Ind.	2018	Darwin	PBMC+serum
LIFT-V 206	Yes	59	M	Non-Ind.	2018	Darwin	PBMC+serum
LIFT-V 211	Yes	40	F	Non-Ind.	2018	Darwin	PBMC+serum
LIFT-V 212	Yes	28	M	Non-Ind.	2018	Darwin	PBMC+serum
LIFT-V 213	Yes	35	M	Non-Ind.	2018	Darwin	PBMC+serum
LIFT-V 218	Yes	35	F	Non-Ind.	2018	Darwin	PBMC+serum
LIFT-V 219	Yes	53	F	Non-Ind.	2018	Darwin	PBMC+serum
LIFT-V 224	Yes	58	M	Non-Ind.	2018	Darwin	PBMC+serum
LIFT-V 227	Yes	38	F	Non-Ind.	2018	Darwin	PBMC+serum
LIFT-V 228	Yes	34	M	Non-Ind.	2018	Darwin	PBMC+serum
S3055	Yes	22	F	Non-Ind.	2018	Launceston	Serum
S3060	Yes	45	F	Non-Ind.	2018	Launceston	Serum
S3063	Yes	41	F	Non-Ind.	2018	Launceston	Serum
S3066	Yes	46	F	Non-Ind.	2018	Launceston	Serum
S3075	Yes	42	F	Non-Ind.	2018	Launceston	Serum
S3078	Yes	43	F	Non-Ind.	2018	Launceston	Serum
S3080	Yes	49	F	Non-Ind.	2018	Launceston	Serum
S4018	Yes	29	M	Non-Ind.	2018	Launceston	Serum
S4020	Yes	27	M	Non-Ind.	2018	Launceston	Serum
S4022	Yes	23	M	Non-Ind.	2018	Launceston	Serum
S4024	Yes	49	M	Non-Ind.	2018	Launceston	Serum
S4030	Yes	24	M	Non-Ind.	2018	Launceston	Serum
S4034	Yes	32	M	Non-Ind.	2018	Launceston	Serum
S4041	Yes	46	M	Non-Ind.	2018	Launceston	Serum

Table S2. Virus strains and abbreviations used in this study.

Subtype	Strain name	HAI/B cell probes	Multiplex
H1N1	A/PUERTO RICO/8/34	PR/1934	
H1N1	A/BRAZIL/11/78	BRZ/1978	
H1N1	A/FUKUSHIMA/141/2006	FUK/2006	
H1N1	A/BRISBANE/59/2007	H1N1 BRIS/2007	
H1N1	A/CALIFORNIA/07/2009	CAL/2009	Cal
H1N1	A/MICHIGAN/45/2015	MICH/2015	Mich
H3N2	A/PORT CHALMERS/1/1973	PC/1973	
H3N2	A/PANAMA/2007/1999	PN/1999	
H3N2	A/BRISBANE/10/2007	H3N2 BRIS/2007	
H3N2	A/PERTH/16/2009	PER/2009	
H3N2	A/VICTORIA/361/2011	VIC/2011	
H3N2	A/TEXAS/50/2012	TEX/2012	
H3N2	A/SWITZERLAND/9715293/2013	SWI/2013	Swi
H3N2	A/NEWCASTLE/22/2014	NCAS/2014	
H3N2	A/NEW CALEDONIA/71/2014	NCAL/2014	
H3N2	A/HONG KONG/4801/2014	HK/2014	HK
H3N2	A/BRISBANE/47/2015	BRIS/2015	
H3N2	A/SINGAPORE/INFIMH-16-0019/2016	SING/2016	Sing
B/Yam	B/MASSACHUSETTS/02/2012	MAS/2012	
B/Yam	B/PHUKET/3073/2013	PHU/2013	Phu
B/Yam	B/SYDNEY/7/2014	SYD/2014	
B/Vic	B/BRISBANE/60/2008	BRIS/2008	
B/Vic	B/BRISBANE/46/2015	BRIS/2015	

Table S3. Flow cytometry staining panels.

**Panel A**				
**Antibody**	**Clone**	**Fluorochrome**	**Catalogue numbers**	**Vendor**
CXCR5	RF8B2	BV421	562747	BD Horizon
LiveDead		Aqua	L34966	Thermo Fisher
CD19	HIB19	BV570	302235	Biolegend
CD24	MLS	BV605	562788	BD Horizon
CCR6	11A9	BV650	563922	BD Horizon
CD20	2H7	BV711	563126	BD Horizon
CD38	HIT2	BV786	563964	BD Horizon
CXCR3	1C6	APC	550967	BD Pharmingen
CD27	O323	AF700	56-0279-42	eBioscience
CD4	RPA-T4	APC-H7	560158	BD Pharmingen
CD8	OKT8	FITC	11-0086-42	eBioscience
CD45	2D1	PerCP-Cy5.5	340953	BD
ICOS	DX29	PE	557802	BD Pharmingen
CD3	UCHT1	PE-CF594	562280	BD
PD-1	EH12.1	PE-Cy7	561272	BD Pharmingen
**Panel B**				
**Antibody**	**Clone**	**Fluorochrome**	**Catalogue numbers**	**Vendor**
IBV HA probes		BV421	N/A	In-house
Live Dead		Aqua	L34966	ThermoFisher
CD3	OKT3	BV510	317331	Biolegend
CD8	RPA-T8	BV510	563256	Biolegend
CD10	HI10a	BV510	301047	Biolegend
CD14	M5E2	BV510	301841	Biolegend
CD16	3G8	BV510	302047	Biolegend
Free SA	N/A	BV510	563261	BD Horizon
CD27	O323	BV605	302829	Biolegend
IgG	G18-145	BV786	564230	BD
IgM	G20-127	BUV395	563903	BD
CD21	B-ly4	BUV737	612788	BD
H3 Swi HA probe		APC	N/A	In-house
CD20	2H7	AF700	560631	BD
H1 Mich HA probe		PE	N/A	In-house
CD19	J3-119	ECD	IM2708U	Beckman
IgD	IA6-2	PE-Cy7	561314	BD

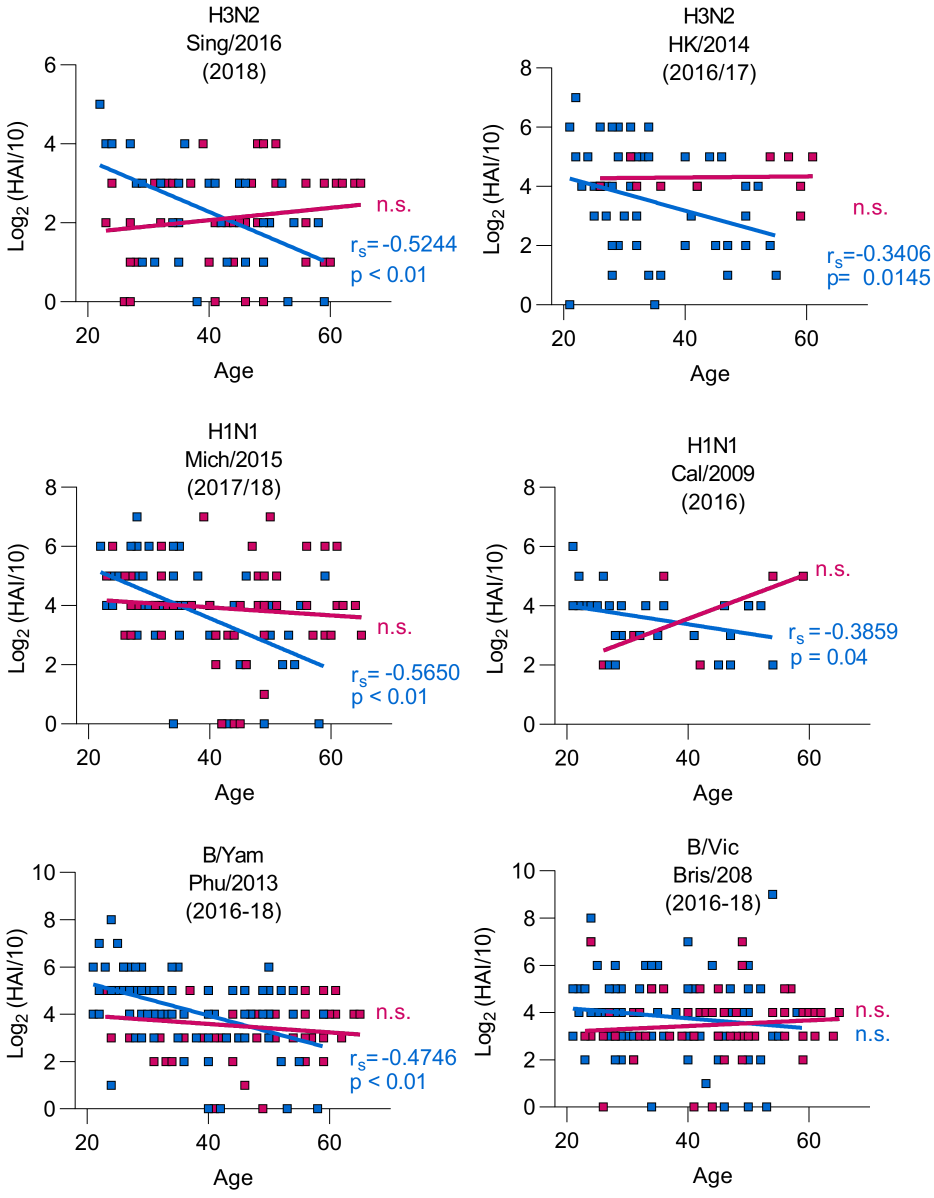

Supplementary Fig 1 Hensen *et al.*

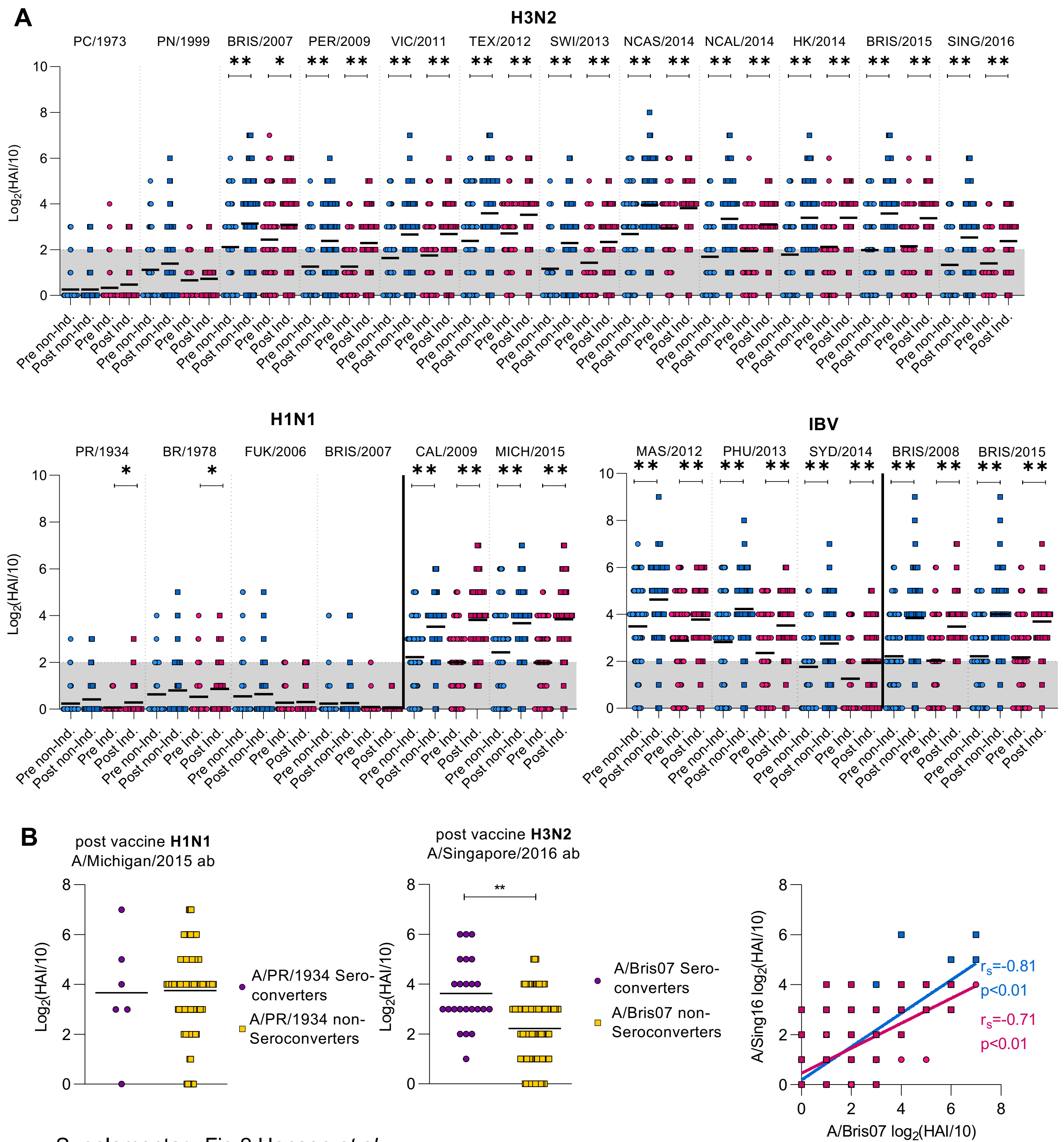

Supplementary Fig 2 Hensen *et al.*

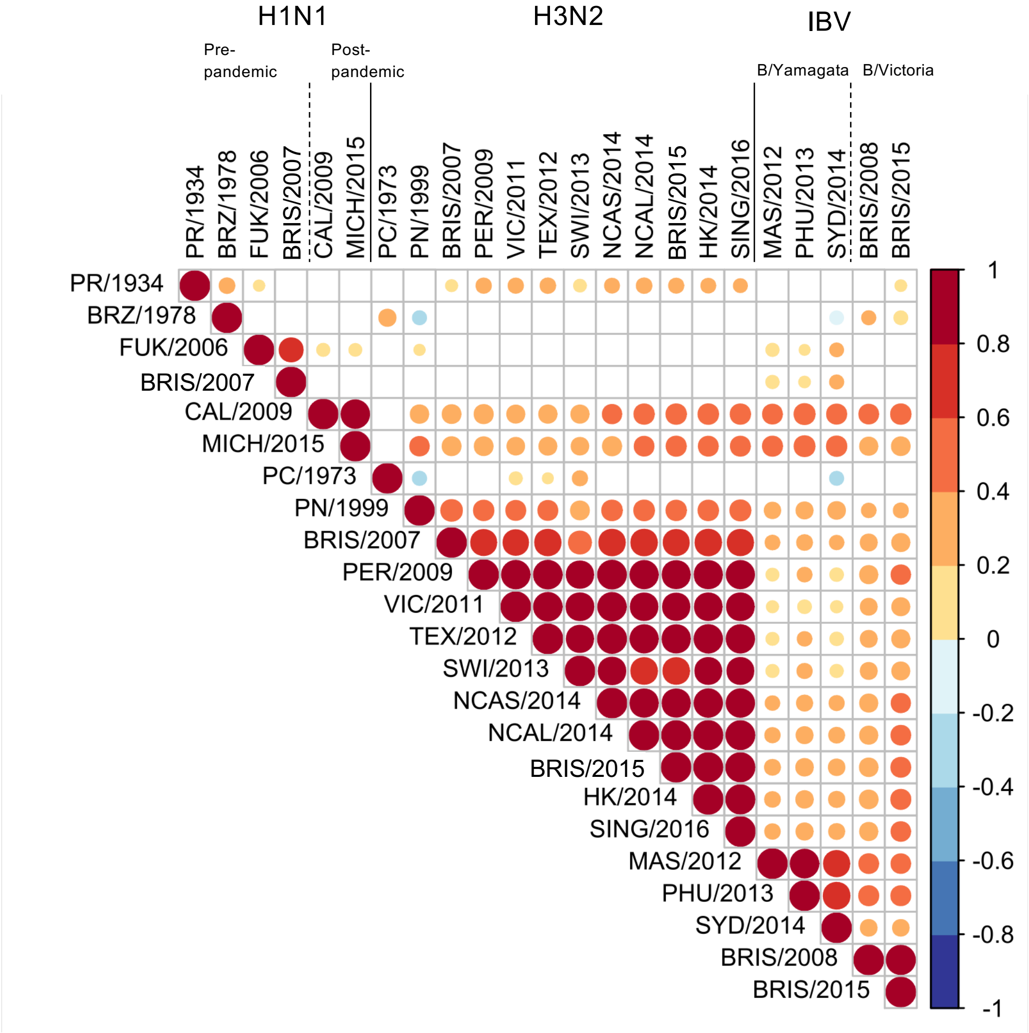

Supplementary Fig 3 Hensen *et al.*

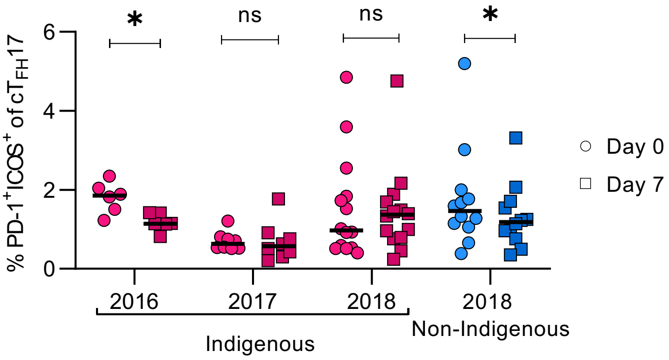

Supplementary Fig 4 Hensen *et al.*

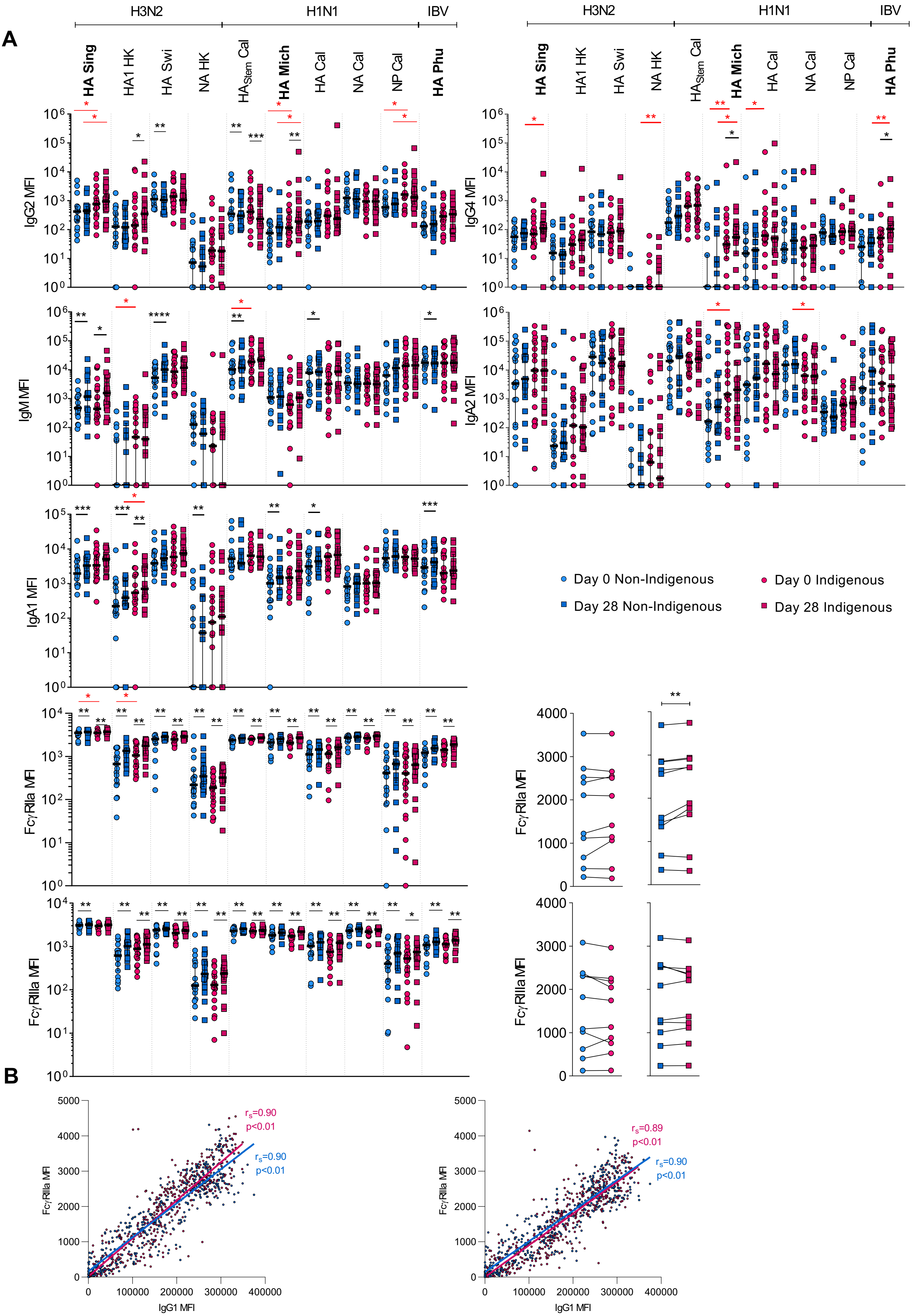

Supplementary Fig 5 Hensen *et al.*

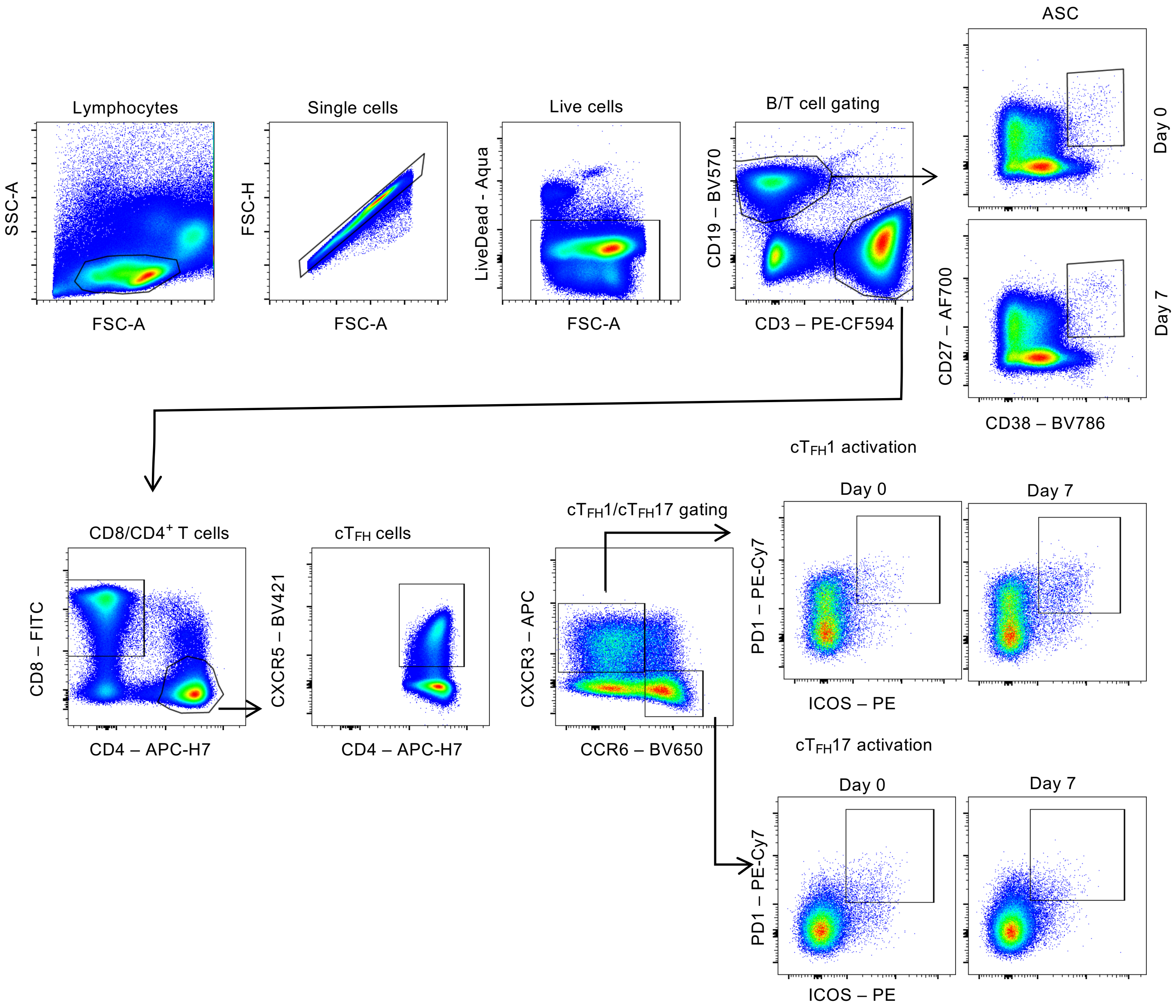

Supplementary Fig 6 Hensen *et al.*

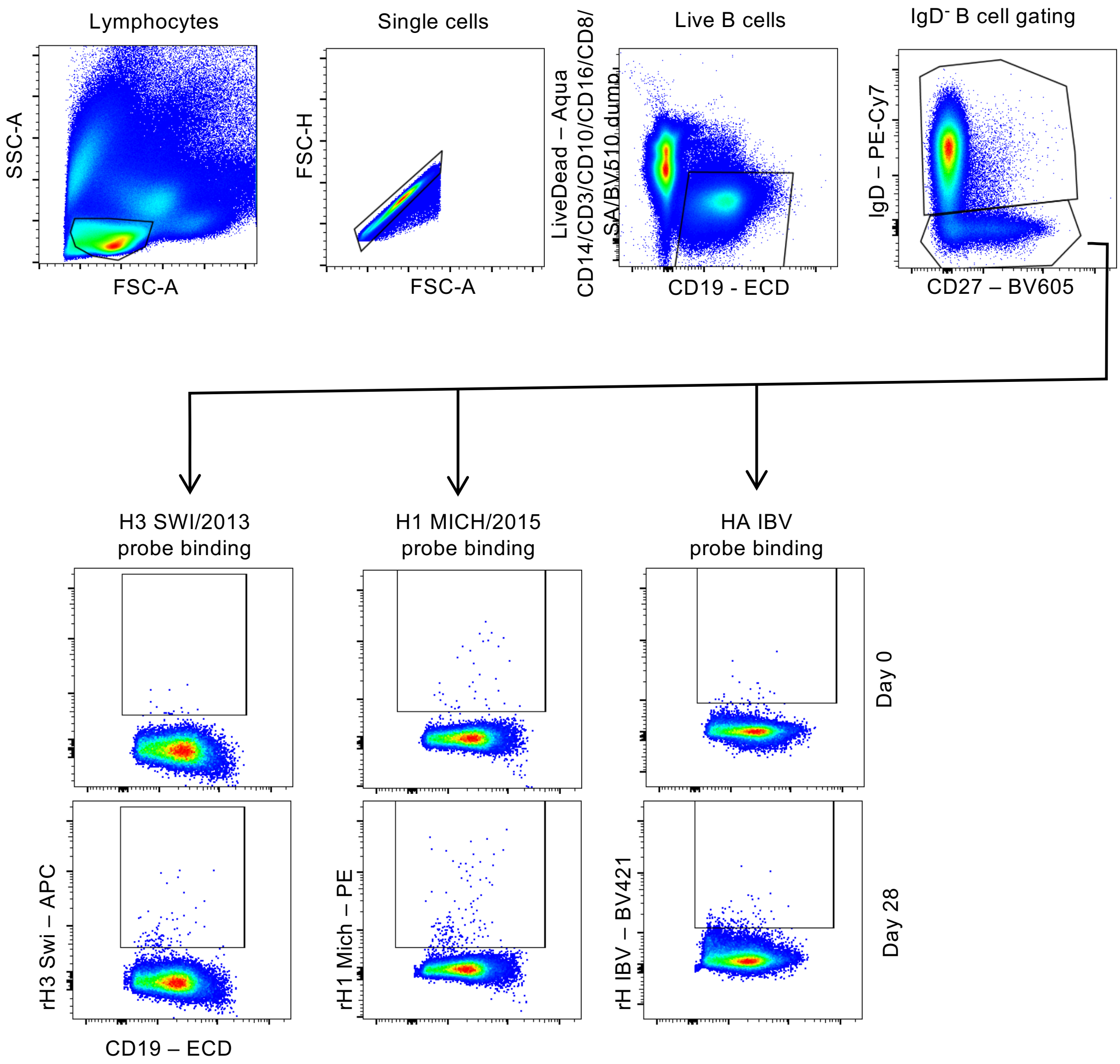

Supplementary Fig 7 Hensen *et al.*
